# Chemical disinfection in healthcare settings: critical aspects for the development of global strategies 

**DOI:** 10.3205/dgkh000371

**Published:** 2020-12-23

**Authors:** Martin Exner, Sanjay Bhattacharya, Jürgen Gebel, Peter Goroncy-Bermes, Philippe Hartemann, Peter Heeg, Carola Ilschner, Axel Kramer, Moi Lin Ling, Wolfgang Merkens, Peter Oltmanns, Frank Pitten, Manfred Rotter, Ricarda Maria Schmithausen, Hans-Günther Sonntag, Kathrin Steinhauer, Matthias Trautmann

**Affiliations:** 1 Institute of Hygiene and Public Health, Bonn University, Bonn, Germany; 2 Tata Medical Center, Kolkata, India; 3 Schülke & Mayr GmbH, Norderstedt, Germany; 4 Departement Environnement et Santé Publique S.E.R.E.S., Faculté de Médecine, Nancy, France; 5 Institute of Medical Microbiology and Hygiene, University of Tübingen, Germany; 6 Institute of Hygiene and Environmental Medicine, University Medicine Greifswald, Germany; 7 Infection Prevention & Control, Singapore General Hospital, Singapore; 8 IKI – Institut für Krankenhaushygiene & Infektionskontrolle GmbH, Gießen, Germany; 9 Hygiene Institute, Medical University Vienna, Austria; 10 Institute of Hygiene and Medical Microbiology, University of Heidelberg, Germany; 11 Department of Hospital Hygiene, Stuttgart Hospital, Stuttgart, Germany

**Keywords:** chemical disinfection, disinfection precautions, disinfection, effectiveness of disinfectants, disinfection process

## Abstract

Chemical disinfection is an indispensable means of preventing infection. This holds true for healthcare settings, but also for all other settings where transmission of pathogens poses a potential health risk to humans and/or animals. Research on how to ensure effectiveness of disinfectants and the process of disinfection, as well as on when, how and where to implement disinfection precautions is an ongoing challenge requiring an interdisciplinary team effort. The valuable resources of active substances used for disinfection must be used wisely and their interaction with the target organisms and the environment should be evaluated and monitored closely, if we are to reliable reap the benefits of disinfection in future generations. In view of the global threat of communicable diseases and emerging and re-emerging pathogens and multidrug-resistant pathogens, the relevance of chemical disinfection is continually increasing. Although this consensus paper pinpoints crucial aspects for strategies of chemical disinfection in terms of the properties of disinfectant agents and disinfection practices in a particularly vulnerable group and setting, i.e., patients in healthcare settings, it takes a comprehensive, holistic approach to do justice to the complexity of the topic of disinfection.

## Zusammenfassung

Die chemische Desinfektion ist ein unverzichtbares Mittel zur Infektionsprävention. Dies gilt für das Gesundheitswesen, aber auch für alle anderen Bereiche, in denen die Übertragung von Krankheitserregern ein potenzielles Gesundheitsrisiko für Mensch und/oder Tier darstellt. Zu erforschen, wie die Wirksamkeit von Desinfektionsmitteln und des Desinfektionsprozesses sichergestellt werden kann und wann, wie und wo Desinfektionsmaßnahmen durchzuführen sind, ist eine ständige Herausforderung, die eine interdisziplinäre Teamarbeit erfordert. Die wertvollen Ressourcen der für die Desinfektion verwendeten Wirkstoffe müssen klug genutzt werden und ihre Wechselwirkung mit den Zielorganismen und der Umwelt sollte genau bewertet und überwacht werden, wenn wir die Vorteile der Desinfektion in zukünftigen Generationen zuverlässig nutzen wollen. Angesichts der globalen Bedrohung durch übertragbare Krankheiten sowie durch neu- und wiederauftretende Krankheitserreger und multiresistente Erreger nimmt die Bedeutung der chemischen Desinfektion ständig zu. Dieses Konsensuspapier befasst sich vor allem mit den entscheidenden Aspekten für Strategien der chemischen Desinfektion im Hinblick auf die Eigenschaften von Desinfektionsmitteln und Desinfektionspraktiken in einer besonders gefährdeten Gruppe und Umgebung, nämlich bei Patienten in Einrichtungen des Gesundheitswesens, jedoch verfolgt es dabei auch einen umfassenden, ganzheitlichen Ansatz, um der Komplexität des Themas Desinfektion gerecht zu werden.

## 1 Introduction

Chemical disinfection is a globally accepted core element in the bundle approach of infection prevention in healthcare facilities and standard precautions [1]. The increase in multidrug-resistant organisms and the threats posed by newly emerging or re-emerging pathogens are being discussed at various levels, including the General Assembly of the United Nations. However, much less attention has been paid to the requirements for the development, marketing and practical application of chemical disinfectant procedures and the consequences for public health from a global perspective. 

During the two-day symposium held by the Rudolf-Schülke-Stiftung, in Hamburg in February 2018, an expert panel of renowned hygienists and infection preventionists, infection control practitioners and microbiologists from Austria, France, Germany, India and Singapore discussed strategies for chemical disinfection with respect to regulations for the approval of chemical disinfectants for application in healthcare settings and its implications for the efficacy of infection prevention. Taking a holistic approach, the discussion also included efficacy testing procedures, predominant active substances for disinfectants, disinfection routines in human medicine, a glance at disinfection in agriculture, as well as risks for the development of tolerances against certain active agents and aspects of toxicity for humans and the environment. 

This paper is based on the Symposium, and while it does not claim to fully reflect the situation in all countries throughout the world, it does present the consensus on items considered to be essential for future global strategies in disinfection and antisepsis, taking into account multinational needs and concerns.

## 2 Definitions

### 2.1 Global variations in the usage of disinfectant and disinfection

For a consensus on global strategies for chemical disinfection in healthcare settings, it is of paramount importance to come to an agreement on how the terms **disinfection** and **disinfectant** are to be defined. When we take a closer look at the usage of these terms, it becomes apparent that a myriad of definitions exist which may resemble each other but which are not completely identical. Directives, laws and ordinances regulating these chemicals use definitions to be followed by manufacturers placing their products on the market, test standards refer to certain definitions, and recommendations and guidelines also have their definitions. Attachment 1 shows examples of definitions and usage for the terms “disinfection” and “disinfectant” and various classification systems for disinfectants in international standards and recommendations. 

Attachment 1 shows examples of different definitions and usage for the terms “disinfection” and “disinfectant” and various classification systems for disinfectants in international standards and recommendations, indicating that comparisons of disinfection practices must be interpreted with caution.

In this context it is important to keep in mind that cleaning generally refers to the removal of foreign material such as soil, dust, and (organic) contaminants using physical/mechanical actions – for example, wiping, applying high-pressure water or air – combined with the chemical action of a surfactant, enzymatic product, or detergent and water. The result is a (usually undefined) reduction in the bioburden and other extraneous chemical substances. The quantitative effect of cleaning has been investigated in several studies, with differing results depending on the process and without exact efficacy requirements published in guidelines. It is considered “an essential step prior to any disinfection process” ([1]).

Major differences exist as to the exclusion or inclusion of sporicidal activity, hand and skin antisepsis, and as to the mode of action described: elimination, removal, killing, inactivation. 

An interesting difference also exists in the explanation of the purpose of disinfection. Some definitions include the aspect “no longer an infection risk”, but many do not, and restrict the purpose to reducing viable microorganisms, without specifying the extent.

### 2.2 Conclusions and consensus 

There is a consensus among this panel that the objective of disinfection is the reduction of the microbial count to the degree needed to interrupt transmission of microorganisms, including bacterial spores and viruses, to prevent infection. 

For this consensus paper, the following definitions apply, although different usages continue to exist in different countries and a consensus has not yet been reached:

- Disinfectants are chemical agents which, when applied to the environment (e.g., air or water), environmental surfaces or animate surfaces (skin or oral mucosa), reduce the load of microorganisms including pathogens, mostly by cidal effects on various parts of the microbial cellular structure (e.g., cell wall, cell membrane, enzymes, DNA, ribosomes, etc.), thereby reducing or possibly eliminating chances of infection and microorganism transmission from the reservoir to another environment or host. If the mode of action is only inhibitory, the risk of development of antimicrobial resistance exists. It should be noted that disinfection can be achieved by physical methods as well.
- Antiseptics are antimicrobials which are used only on animate surfaces such as skin, mucous membranes, eye, and wounds.

In some countries, a differentiation is made between the terms “skin disinfection”, referring to intact skin, and “skin antisepsis”, referring to measures applied to damaged skin. In many countries, the terms “hand disinfection” or “hand rub” are used for “hand antisepsis”.

A globally accepted glossary for the definitions of terms relating to antisepsis, disinfection and sterilization for all areas of application is highly recommended.

This paper on developing global strategies of disinfection deals with disinfection procedures, including hand and skin antisepsis (more specifically, preoperative surgical site skin antisepsis), according to the definition above. In particular, it deals with hand and skin antisepsis, surface disinfection, disinfection of medical devices and water. The authors of this paper believe that it is imperative to address and clarify disinfection issues at a global level. 

## 3 Regulatory affairs and test standards for disinfectants

Although disinfectants are just one constituent in the disinfection procedure, product quality and the choices available to the user in the individual countries also have a large impact on the actual practice and effectiveness of disinfection.

The legal regulations pertaining to the marketability of disinfectants differ considerably throughout the world. Whereas some countries do not have any regulatory body for the approval of disinfectants in hospitals, some countries have a very complicated structure for registration and efficacy testing of disinfectants and antiseptics for various applications.

Microbicide formulations may be classified as *medicinal products*, e.g., hand and skin antiseptics, as *medical devices* if an instrument or surface disinfectant has been declared for use to disinfect a specific medical device, as *biocidal products* or as *antimicrobial pesticides* [2], or they may have *dual claims* (medical device and biocidal product). It would be beyond the scope of this paper to describe all systems presently in place; thus, two widely acknowledged systems are presented briefly by way of example.

### 3.1 Disinfectant categories in Europe

#### 3.1.1 Biocidal products 

Approval of biocidal products is regulated by Regulation (EU) 528/2012 ([3] Biocidal Products Regulation (BPR), cf. Attachment 1). Within this scheme, biocidal products are divided into four main groups. Disinfectants fall into main group 1 “Disinfectants and General Biocidal Products”. In this group, disinfectants are further divided into 5 product types (PT):

- PT 1: Human hygiene (biocidal products applied on or in contact with human skin, such as hygienic handrubs, “with the primary purpose of disinfecting the skin or scalp”)
- PT 2: Disinfectants and algaecides not intended for direct application to humans or animals (e.g., surface disinfectants, textile disinfectants, disinfection of air, water not used for human or animal consumption Please note: this product type includes various categories of intended use of medical [e.g., hospitals] and non-medical applications with different testing requirements and pass criteria, and it also contains specific uses such as room disinfection with vaporized biocides)
- PT 3: Veterinary hygiene
- PT 4: Food and feed area
- PT 5: Drinking water (please note: in many European countries there are special, stricter requirements for the microbiological quality of water used in healthcare facilities).

The EU Regulation prescribes a two-step registration procedure for biocidal products, which consists of the approval of the active substance and the approval of the biocidal product. The European Chemicals Agency (ECHA) is the European regulatory authority for implementing this legislation. Once an active substance has been approved and included in the “Union List”, manufacturers have to apply for registration of their products containing these substances within a predetermined time frame. The requirements for manufacturers authorizing their products in accordance with the EU’s legislation are specified by the Biocidal Products Regulation. Each of the 28 EU member countries has its “Evaluation Competent Authority” which is responsible for the national biocidal product authorisation. Switzerland, although not a member of the EU, also complies with the EU regulations for biocidal products. The biocide active substance review program will be completed by the end of 2024. 

Approval is based on European efficacy test standards (CEN) for disinfectants and antiseptics. When no European standards are available for specific applications, other test methods issued by national bodies such as VAH (Verbund für Angewandte Hygiene / Association of Applied Hygiene), DVG (Gesetz für eine bessere Versorgung durch Digitalisierung und Innovation (Digitale-Versorgung-Gesetz)), AFNOR (Association Française de Normalisation), US-EPA (U.S. Environmental Protection Agency), AOAC International (Association of Official Analytical Collaboration International) or ASTM International (American Society for Testing and Materials) are available and may be used. Where no test standards are available, suitable test protocols may be designed by the applicant phases (cf. ECHA [4], VAH methods [5]). 

The EN methods for efficacy testing are based on the tiered approach in three phases:

- Phase 1: Preliminary in-vitro laboratory screening tests (basic suspension tests).
- Phase 2, step 1: Quantitative suspension tests under simulated practical conditions.
- Phase 2, step 2: Quantitative laboratory tests under simulated practical conditions to test disinfectant activity when applied to a carrier or living tissue (hard or porous surfaces, skin).
- Phase 3: Actual field trials under practical conditions (first studies have recently been published with regard to hand antisepsis [6]).

This approach pertains to PT 1 and PT 2 products and includes claims for the activity spectra bactericidal, mycobactericidal, tuberculocidal, sporicidal, yeasticidal, fungicidal, virucidal (enveloped/non-enveloped), depending on the intended field of application. According to the ECHA Guidance on the Regulation, for general applications in the medical sector, PT 2 products should be at least sufficiently effective against bacteria and yeasts ([4], p. 67). ECHA-approved active substances and biocidal products are published online [7]).

Current national approval and certification procedures as well as efficacy tests were established in some European countries many decades prior to the European regulations:

- Germany (VAH),
- Austria (ÖGHMP),
- France (Société française d'Hygiène Hospitalière).

The Association for Applied Hygiene (VAH e.V.) in Germany develops efficacy test methods, performs post-marketing surveillance, issues certificates for tested disinfectants and publishes the VAH List of Disinfectants [8]. This list contains detailed information on active ingredients/substance class, activity spectrum, contact times and use concentrations of VAH-certified disinfectants, all of them suitable for disinfection in medical and/or care settings. Test methods for virucidal activity and test methods for disinfectants to be used in veterinary, food and feed areas are developed by and in cooperation with two other professional societies, the German Association for the Control of Viral Diseases (DVV) [9] and the German Society for Veterinary Medicine (DVG), which also publishes lists for disinfectants for their scope (http://www.desinfektion-dvg.de/index.php?id=1789). 

In view of the legally binding status of ECHA approval for disinfectants, it is presently unclear how the national certification and listing processes of VAH in Germany (or, e.g., ÖGHMP in Austria) will develop in future. 

#### 3.1.2 Drawbacks of the ECHA regulations 

As noted above, the definition for disinfection (or disinfectant) is usually merely technical, without epidemiological evidence, and does not necessarily include the aspect of “maintaining health”. When we postulate that disinfection is performed in order to prevent the transmission of pathogens and/or reduce the viable microbial count to a degree which ensures safety and well-being for people and animals in terms of the prevention of infection and/or the prevention of spread of infection, then the aspect of “maintaining health” is seen as the top priority when weighing benefits and risks of the disinfection and antisepsis process. 

Infectious doses vary between pathogens, and the extent of contamination also varies by several decimal powers. Consequently, it is not only the in-vitro efficacy of a disinfectant which decides how effective disinfection is. An equally important aspect is the standardization of the complete disinfectant procedure itself, which is often insufficiently taken into account, especially in those instances where disinfection is a manual procedure (e.g., surface disinfection [10]). 

It was also reported that although pathogens using local action have lower infective doses, they are not less virulent than those using distant action. It was also found that virulence was negatively correlated with infective dose and higher in pathogens infecting wounded skin, compared with those ingested or inhaled [11], [12] (Table 1 (Tab. 1)). 

Apart from testing biocidal activity, the ECHA approval process assesses the potential toxic effects of biocidal products on human and animal health or the environment. It does not, however, take into account specific needs in terms of local or regional specific infection risks (e.g., activity against *C. difficile*) or outbreak control, neither does it consider long-term sequelae as a consequence of overusing certain substances when others are not available. The focus is on safe handling and usage of biocidal products rather than preventing health hazards by means of biocidal products. Thus, an active substance which has proven to be very effective against certain target organisms may not be approved because ECHA considers the (toxic) risks to outweigh the benefits, and deems other substances with less toxic risks to be equally effective. As an example, the active substance glutaraldehyde is considered as a candidate for substitution by ECHA, and consequently the competent authority shall perform a comparative assessment as part of the evaluation of an application for biocidal product authorization. This also means that the approval as an active substance has to be renewed within 5 years. Restrictions on the use of glutaraldehyde in European countries are likely. 

Ethanol may possibly be classified as carcinogenic, independent of the exposure route. Its approval for PT1 (product type 1) is still pending. The warning *“May cause cancer”* will then have to be printed on the label of handrubs containing ethanol, which will most likely considerably reduce the acceptance of products containing this well-established active substance by healthcare personnel. This would be despite the fact that the overall dermal absorption of ethanol, even given excessive use of ethanol-based disinfectants, is below toxic levels in humans [13]. In general, the diversity of active substances available for the manufacture of disinfectants to be used for medical indications and for niche medical applications is expected to decrease drastically within the scope of ECHA regulations. 

Furthermore, the approval of active substances by ECHA is an extremely costly and time-consuming process for disinfectant manufacturers. Consequently, manufacturers are less inclined to invest in research for novel substances or innovative procedures, smaller manufacturers may not survive, and less effective (but safer to use) substances in sublethal (bacteriostatic) dosage may increasingly enter the market. Since ECHA regulations have an impact on regulations in other countries, the problems described are not only problems on a European scale but on an international scale as well.

### 3.2 Borderline classification: medicinal product, medical device, biocidal product 

The discussion of distinguishing medicinal products from medical devices is relevant for those countries in which hygienic handrubs and skin antiseptics are officially registered as medicinal products (pharmaceutical drugs) as is the case in Germany, India, the U.S. or Australia, for instance.

In Europe and the U.S., medicinal products and medical devices are regulated by separate laws which cannot be applied simultaneously. Because dual labeling is generally not accepted by drug administration agencies, these products are regulated by either the medical device or the drug legislation, but not both. Medicinal products are distinguished from medical devices by their pharmacological, metabolic, and/or immunological effects, while the mode of action of medical devices is predominately based on physical aspects [14].

The intention of preventing or treating illness may apply to both medicinal products and medical devices, and therefore does not qualify as a distinguishing characteristic between the two. Thus, the only reason to objectively differentiate between a drug and a medical device is its pharmacological, immunological or metabolic effect, compared with the physical effect of the medical device, which may merely support pharmacological, immunological or metabolic effects.

The European MEDDEV-Borderline Guideline [15] defines a pharmacological effect as an interaction between molecules of the substance in question and a cellular component, such as a receptor, which either elicits a direct response or blocks another one in response to a third agent. The view therefore does not specifically demand interaction of substance molecules with ‘cellular components of the host’, but merely requires ‘cellular components’. A more logical European Court ruling (Judgment of the Court (Fourth Chamber) of 10 July 2014, [16]) supports the definition of a pharmacological effect of a substance as an interaction with any cellular components within the host’s body, including foreign targets cells like bacteria, viruses, or parasites. This opinion is also supported by the European Directive 2004/27/EG, a revised version of the Directive 2001/83/EG section 2, subsection 2, which elevates the MEDDEV-Borderline guideline to the instrument of choice in cases of uncertainty for defining a new technology.

Another borderline situation exists with respect to a potential dual-use classification as medicinal product and as a biocidal product. The existence of multiple test protocols and classification options for handrubs for medical purposes raise a number of questions and are still under debate in Europe. 

Products specifically intended for the cleaning, disinfection or sterilization of medical devices which are to be used for diagnostic and/or therapeutic purposes for human beings generally fall under the scope of the European Medical Device Regulation (MDR 2017/745/EU, last updated 2017 [17]). However, dual-use claims are possible. This means that surface disinfectants may be classified as a biocidal product and/or as a medical device at the same time. Products intended for disinfecting surfaces of medical devices and their accessories must be registered as medical devices if their application is explicitly claimed for a specific medical device [17], [18]. In practice, a disinfectant with a dual registration can thus be used for surfaces such as floors as well as for the disinfection of medical devices.

### 3.3 Disinfectant categories in the U.S.

In the U.S., manufacture and sale of disinfectants (referred to as antimicrobial pesticide products) are regulated by the Environmental Protection Agency (EPA) under the statutory authority of the *Federal Insecticide, Fungicide and Rodenticide Act* (FIFRA). Documents from “Series 810” specify the requirements for EPA registration ([19] cf. Attachment 1). It is possible to apply for public health claims and non-public health claims. Specific test guidelines exist, for instance to support a specific claim for surface disinfectants such as limited-spectrum disinfectant, general or broad-spectrum disinfectant, and hospital or healthcare disinfectant for hard nonporous surfaces with fungicidal and virucidal claims, for tuberculocides and sporicides. The test methods used to evaluate these efficacy claims of disinfectants in the U.S. are mainly developed by the Association of Official Analytical Chemists (AOAC International).

There are different test methods for water-soluble powders and liquids, sprays and towelettes. “Hospital grade” disinfectants must demonstrate effectiveness against two pathogens, *S. aureus* and *P. aeruginosa* in three production lots to meet registration requirements. The Office of Pesticide Programs (OPP) reviews the efficacy data prior to EPA registration. Many products approved for use in hospitals may also be approved for use in homes. The lists of EPA-registered disinfectants are available from the website http://www.epa.gov/oppad001/chemregindex.htm. Users are obligated to read the label and technical data sheets of EPA-registered disinfectants to obtain detailed relevant information on the intended use and activity spectrum and must follow these instructions [20]. 

Presently, the EPA is developing a strategy for risk-based post-registration testing to ensure the effectiveness of hospital-level disinfectants [21]. 

Liquid sterilant chemicals and high-level disinfectants intended for use on critical and semi-critical medical/dental devices and instruments as well as hand antiseptics (handwash or handrub agents) are regulated by the Food and Drug Administration (FDA). They are tested for their specific claims with ASTM International (formerly American Society for Testing and Materials) standards. 

### 3.4 Disinfectant categories in ASEAN and India 

For Asia, ASEAN, the *Association of Southeast Asian Nations*, agreed on a joint ASEAN Medical Device Directive [22]. It includes medical devices intended to be used for sterilizing medical devices, or disinfecting as the end point of processing. One example of implementation into national law is the Medical Device Guidance issued by Singapore’s Health Science Authority. An ASEAN biocide regulation does not exist. FDA and/or EPA registration, availability, affordability, and the properties of individual active agents are taken into consideration.

In India, handrubs are regulated by the Indian Pharmacopeia Commission ([23] cf. Attachment 1). A regulatory body for disinfectants and biocidal products does not (yet) exist in India. The choice varies considerably depending on the hospital. The Infection Control Team often solely relies upon the literature provided by the manufacturers of disinfectants [24]. In 2015, National Guidelines for Clean Hospitals, applicable to tertiary care hospitals were published ([25] cf. Attachment 1). These guidelines include a list of hospital-grade disinfectant substances for use in all healthcare settings such as alcohols (60–90% ethyl or isopropyl alcohol), chlorines, phenolics, quaternary ammonium compounds, iodophors and hydrogen peroxides, but no commercially available products.

India has its own Medical Devices Rules (2017), issued by the Drugs Controller General, which took effect on 1 January 2018 to regulate medical devices, including disinfectants to be used on medical devices [26]. India-specific disinfectant testing standards include the Indian Standard for Disinfectant Fluids, Phenolic Type – Specification (BIS IS 1061:2017) [27].

In this context, it is important to point out that restrictions on the use of certain disinfectant and antiseptic agents may vary depending on the country and the time period. For example, the National List of Essential Medicines of India (2011) [28] and the National Formulary of India [23] include formaldehyde and glutaraldehyde as disinfectants.

The central government of India designated five laboratories having facilities for carrying out tests and evaluations of medical devices as Central Medical Device Testing Laboratories under MDR 2017 for the purposes of (a) testing and evaluation; (b) functioning as an appellate laboratory; and (c) carrying out any other function which may be specifically assigned to it by the Central Government in relation to the medical devices. The designated laboratories are accredited by the NABL (National Accreditation Board for Testing and Calibration Laboratories). The Central Drugs Laboratory (CDL), Kolkata, is the national statutory laboratory of the Government of India for quality control of Drugs and Cosmetics and responsible for testing disinfectants (medical devices), surgical dressings and others [29]. 

### 3.5 Conclusions and consensus

There is a general tendency to have separate national regulations for disinfectants as medical devices, for hand and skin antiseptics and for disinfectants for inanimate objects (surfaces, equipment). Most regulations differentiate between the intended scope healthcare or consumer products, as well as veterinary use, food and feed area, agriculture and water. 

At the same time, the assessment of safety for the user and the environment are increasingly gaining importance. Since on the one hand disinfectant and antiseptic agents cannot be completely harmless to humans as a consequence of their inherent microbicidal properties, but on the other hand their application is absolutely indispensable in order to interrupt the spread of (multiple-resistant) pathogens and control outbreaks, the benefit:risk ratio for each individual active substance has to be carefully weighed. 

There is a consensus among this panel of experts:

- Reliable quality of disinfectants must be ensured for disinfectants in all settings where disinfection is indicated.
- An international agreement on major criteria for the classification of disinfectants (and antiseptics) and their respective regulations should be reached.
- An international agreement on major criteria for efficacy testing reflected in these regulations should be reached.
- The risk-benefit analysis of authorization and regulation procedures for biocidal agents should take sufficient account of the antimicrobial efficacy aspects.
- Flexibility within regulations is necessary to allow for (changing) local and regional specific infection control needs.
- On-site manufacturing/preparation of disinfectants should be performed with standardized processes, and the processes and results should be monitored.
- Standardized efficacy testing of disinfectants and antiseptics should be required prior to sales.
- Government regulations for placing disinfectants on the market should be available for all disinfectant categories.
- Postmarketing surveillance of disinfectant and antiseptic products should be performed by an independent body.

## 4 Active substances used for disinfection

### 4.1 Active substances used in Europe

#### 4.1.1 Overview of active substances for disinfectans used in Europe

As of 8 November 2019, the ECHA lists 11 active substances to be approved for application as biocides for “human hygiene” (product type 1), which means for direct application on humans (skin), from various membership countries (cf. Table 2 (Tab. 2), [https://echa.europa.eu/de/information-on-chemicals/biocidal-active-substances]). 

Table 3 (Tab. 3) shows the status of approval for disinfectants and algaecide not intended for direct application on humans or animals (product type 2, including surface disinfectants). The majority of substances are still under review. Products containing substances which are still under review can be marketed until 31 December 2024.

Open-access reports or data of the actual quantities of disinfectant substances sold and used in healthcare settings in Europe from independent sources are not available. According to a Market Data Forecast report released in October 2018, the most frequently employed substances for surface disinfectants in Europe are quaternary ammonium compounds [30]. The growth in this category is said to be a result of widespread availability, greater consumption of surface disinfectants in hospitals, and low prices. Overall, liquids are more widespread than wipes and sprays. It should be noted that especially sales of surface disinfectants continue to increase in all sectors, including the healthcare sector. 

A questionnaire survey conducted by EUNETIPS on cleaning and disinfection in European hospitals published in 2011 showed that alcohols were the most frequently used chemical for disinfection in this setting, followed by oxidants and halogens [31]. Quaternary ammonium compounds were not included in the list of possible answers to the question “What do you use for chemical disinfection?” at that time. The majority of countries stated that they use mops, cloths or both with water and detergent (soap) for cleaning.

Alcohol-based handrubs for hand antisepsis containing ethanol, propanol or isopropanol are the gold standard. Povidone-iodine-alcohol seems to be the most frequently used agent for preoperative skin antisepsis in European hospitals, but chlorhexidine-alcohol or octenidine-alcohol preparations are also widely used throughout the world [32]. 

#### 4.1.2 Active substances for disinfectants used in Germany

A look at the database of tested and certified chemical disinfectants issued by the Association of Applied Hygiene (VAH) in Germany reveals more details on the currently prevailing substances marketed in Germany for medical and care settings (including public healthcare, Table 4 (Tab. 4)). (Note: These substances might change by 2024 depending on ECHA approval.)

Table 5 (Tab. 5) gives an overview of the main basic active substances of VAH listed disinfectants sorted by field of application (sorted by frequency). 

### 4.2 Active substances of FDA- or EPA-registered hospital disinfectants 

The National Pesticide Information Center's portal Product Research Online (NPRO, http://npic.orst.edu/NPRO/) allows the search for pesticides by type (e.g., biocide, disinfectant, sterilant, sporicide, virucide), product formulation and toxic signal words, and filter the results for products with active EPA registrations. It is also possible to sort by very specific use sites and pest type. The website is updated weekly. Typically, products for low-level disinfection will contain quaternary ammonium compounds (quats), sodium hypochlorite or phenolics, also 70–90% ethyl or isopropyl alcohol and iodophors, depending on the field of application. Intermediate-level hospital disinfectants will typically include 70–90% ethyl or isopropyl alcohol, iodophors, sodium hypochlorite, improved hydrogen peroxide or phenolics as active substances [19], [33]. 

A look at the FDA-approved sterilants and high-level disinfectants on the internet [34] with general claims for processing reusable medical and dental devices (as of September 2015) reveals that the most common active ingredient in the 36 listed products is glutaraldehyde in concentrations between 2.4% and 3.4%, also in combination with alcohol or phenolics, followed by orthophthaldehyde (OPA), hydrogen peroxide and peracetic acid. 

### 4.3 Special notes on active substances by category

#### 4.3.1 Alcohol-based handrubs (ABHR)

According to the WHO Hand Hygiene Guideline, handrub formulations should be alcohol-based and meet either European standards (EN) or ASTM (American Society for Testing and Materials) standards [35]. 

Although ABHR are viewed as the agent of choice for hand hygiene in healthcare in most countries, over-the-counter (OTC) products with inadequate efficacy are sometimes used, or plain or medicated soap is used in situations where handrubs should be employed. 

The FDA differentiates between health care and consumer antiseptics. In April of 2019, FDA finalized the 2016 Consumer Antiseptic Rub proposed rule on the safety and effectiveness of consumer antiseptic rubs and topical antimicrobial drug products for over-the-counter human use. It deferred further rulemaking on the three active ingredients benzalkonium chloride, ethanol, and isopropanol, which are the only active ingredients eligible for evaluation under the OTC Drug Review for use in OTC consumer antiseptic rub products. There are 28 ineligible active ingredients listed, among them are benzethonium chloride, chlorhexidine digluconate, hexachlorophene, methylbenzenthonium chloride, phenol and triclosan [36], [37]. 

As stated above, ECHA has approved 1-propanol and 2-propanol, but not yet ethanol as a substance permitted for PT 1 use (biocidal products applied on or in contact with human skin such as hygienic handrubs). Alcohol-based handrubs, containing ethanol or 1-propanol or 2-propanol as their main active substances are the gold standard for handrubs in Europe. For example, the German KRINKO recommends the use of alcohol-based products without any other additives such as chlorhexidine (CHG) or mecetronium etilsulfat. Concern as to resistance development of certain bacterial strains to chlorhexidine is increasing, e.g., CHG resistance may be detected in multi-resistant isolates such as extremely drug-resistant *Klebsiella pneumoniae* [38], [39], [40], [41], [42]. 

#### 4.3.2 Skin antiseptics for preoperative surgical site preparation

WHO global guidelines and the German KRINKO recommend alcohol-based agents with remanent antiseptic additives such as CHG or octenidine dihydrochloride or the combination of povidone-iodine with alcohols for surgical site preparation of intact skin [43], [44]. The 2017 CDC Prevention Guideline for the Prevention of Surgical Site Infection recommends that preparation in the operating room should be performed using an alcohol-based agent unless contraindicated [45].

Based on a systematic review of current literature [46], WHO officials state that where these preparations are not available or too costly, local production is possible and should be encouraged.

#### 4.3.3 Instrument disinfectants

Active substances listed as “commonly-used chemical disinfectants” for item or equipment disinfection in the WHO Guidelines for Decontamination and Reprocessing of Medical Devices [47] include ortho-phthalaldehyde, glutaraldehyde, formaldehyde, peracetic acid, hydrogen peroxide, chlorine-based compounds (sodium hypochlorite), alcohol and chlorine dioxide. 

#### 4.3.4 Surface disinfectants

A recent *international* survey of cleaning and disinfection practices in the healthcare environment [48] indicated that, on a global scale, halogens (82%) were by far the most frequently used substance group, followed by alcohols and quaternary ammonium compounds. Their survey included 110 healthcare professionals, representing 23 countries (with North America, South America and Africa being underrepresented). 

### 4.4 Conclusions and consensus

There is a consensus among this panel of experts:

- It must be ensured that the variety of active substances available for disinfectants is sufficient to be able to choose the adequate agent based on a hygienic-medical risk analysis for the benefit of patients and staff safety.
- Wide availability of active substances/supplies should be supported by national governments.
- Local conditions for storage must be taken into account (high temperatures, no air-conditioning).
- The actual consumption/sales of active substances and combination of substances should be open-access published and analyzed according to its intended scope (medical facilities, veterinary medicine, agriculture, drinking water etc).
- More studies are needed with respect to the efficacy of chemical disinfectants and active substances against certain (multidrug-resistant Gram-negative) bacterial strains.
- Restricted efficacy of quaternary ammonium compounds against *C. difficile* must be noted.
- Disinfectant lists (with information on the active ingredients, contact time and concentration, and activity spectrum) are helpful for selecting disinfectants.

## 5 Disinfection practices in human healthcare settings

For hospitals, a myriad of recommendations and guidelines exist for chemical disinfection practices; however, there are much fewer with reference to other medical care settings, such as resident doctors and dentists, outpatient clinics, rehabilitation centers and others. 

### 5.1 Hand hygiene 

Hand hygiene has been the main focus of national and international guidelines, recommendations and campaigns for infection prevention. Hand hygiene encompasses handwashing, hygienic handrub, surgical handrub, glove wearing and skin care. Most national and international guidelines today recommend alcohol-based handrubs for hygienic hand antisepsis (cf. chapter 4.3.1 on active substances) and define their indications for hand hygiene based on or similar to the 5 moments for hand hygiene according to WHO. This does not mean, however, that actual hand hygiene practice is the same everywhere. 

#### 5.1.1 Hand antisepsis or hygienic handrub (or disinfection)

In 2013, the European Centers for Disease Control (ECDC) published a point-prevalence survey of healthcare-associated infections and antimicrobial use in European acute care hospitals 2011–2012, which included alcohol-based handrub consumption as a proxy indicator for hand hygiene. The active ingredients and their concentrations in these handrubs were not specified. The ECDC evaluated data from 820 hospitals in 31 countries. The median consumption varied widely between countries from 10 l/1,000 to 50 l/1,000 patient days (cf. [49], therein Figure 16, distribution of the consumption of alcohol handrub by country, ECDC PPS 2011–2012). However, the ECDC points out that the data must be interpreted with caution due to a number of methodical problems and the fact that the PPS sample was not representative for 8 (24%) countries ([49], p. 12).

For local production of alcohol-based handrubs (ABHR) in situations where commercial products are either not available or too costly, the WHO recommends two formulas containing either ethanol (80% v/v) or isopropanol (75% v/v) as their main active substances as well as 0.125% (v/v) hydrogen peroxide and 1.45% (v/v) glycerol. Although feasibility and successful implementation of local production has been demonstrated in many places, problems continue to exist with regard to acceptance (smell), procurement of dispensers and ingredients, and quality control measures for production [50]. 

Handrubs may come in a variety of delivery systems: liquids, foam, gel, and wipes. Gel formulations may constitute a problem in tropical countries with high ambient temperatures and humidity. Some consumers may favor hand antisepsis with wipes, and some studies have shown positive results for virus reduction when wipes are used [51]. However, efficacy tests conducted to evaluate alcohol-based wipes for hand antisepsis indicated that safe evaluation of wipes with a test protocol designed for liquids is not possible. Wipes deliver significantly less active substance than conventional liquid handrubs, and may result in insufficient disinfectant wetting of the hands. 

Shorter contact times of 15 s instead of 30 s, which is the usual duration, are sometimes also considered an option to enhance compliance and save time. A prospective, randomized crossover study showed that hands may be wet within 15 s [52]. An experimental study employing the WHO technique of handrubbing with 3 ml ABHR with different durations demonstrated that 15 s was not inferior to 30 s in reducing bacterial counts [53]. In addition, other studies in which application time was reduced to 15 s showed a similar effect when compared to 30 s of handrubbing [6], [54]. 

Thus, recent studies have investigated application volumes of alcoholic handrubs as a function of time. In a study examining dosages for alcoholic handrubs, Wilkinson et al. found that the optimum volume in terms of user acceptability was 1.5–2 mL, resulting in a drying time of 20–30 s [55]. However, earlier studies targeting antimicrobial efficacy demonstrated that volume plays an important role, and that the applied volume of alcoholic handrubs should not fall below 3 mL to ensure microbiocidal efficacy as stated in EN 1500 [56]. 

In their investigation on shortening the application time of alcohol-based handrubs to 15 s, Kramer et al. suggested that reducing application time may improve hand hygiene compliance in clinical practice [54]. In that study, EN 1500:1997 [57], which is based on Wilcoxon statistics and request a total of 12–15 test subjects, was modified. The reference procedure based on 60% (v/v) Propan-2ol was applied only once with 1x 3 mL for 30 s contact time, instead of 2x3 mL for 2x 30 s, as requested by EN 1500:1997. 

In the latest version of this method (EN 1500:2017), a statistical evaluation based on the hypothesis of inferiority (Hodges-Lehmann) is used [58]. A total of 18–22 test subjects are included in the tests, where a handrub formulation is tested based on a standard handrub protocol and compared to the reference procedure. The reference procedure includes application of 60% (v/v) Propan-2ol for 2x 30 s. In order to ensure reproducibility and standard conditions for the safe evaluation of handrubs, this standard only allows 30 s as the minimum contact time. According to EN 1500:2017, a handrub which has fulfilled the requirements (i.e., procedure with product shall not be inferior to procedure with reference product propan-2-ol) is deemed suitable to be used as medical hygienic handrub. 

As time pressure and workload are thought to impact compliance in the healthcare setting, it is understandable that a shortened contact time is desirable in practice. However, in selecting a safe product, this should only be taken into consideration if handrubs successfully completed the standard test protocol as defined by EN 1500:2017 for 30 s versus the reference applied for 2x 30 s with 2x 3 mL. Additionally, if measures such as shortening the contact time are being considered, this should be validated under practical conditions in the respective healthcare setting to ensure not only application of safe alcohol-based handrubs, but also safe processes in infection prevention [59]. 

#### 5.1.2 Surgical handrub

The immediate efficacy of alcohol-based hand antiseptics is impaired by residual moisture, for instance, resulting from a preceding handwash [60].

Therefore, hands should not be routinely washed just prior to antisepsis unless there is a good reason for it, such as visible soiling. Even after carefully drying the hand, residual moisture may remain on hands for up to 10 minutes. Thus, the time between handwashing and disinfection should be longer than 10 minutes. In surgical operations following the first operation of the day, hand washing may be omitted. A shortened application time (1.5 minutes) is equal to 3 min in terms of efficacy. Hands should be air dried before gloves are put on, otherwise the perforation rate of gloves will increase. Disinfection efficacy is significantly higher when hands are allowed to dry for 1 minute after the washing phase and before the disinfection phase [60]. 

#### 5.1.3 Conclusions and consensus

There is a consensus among this panel of experts:

- Alcohol-based handrubs without additives with sustained efficacy are the agent of choice for hygienic and surgical handrubs.
- The addition of sustained effective additives (such as 2.5% chlorhexidine) is under debate in some countries, because the efficacy is the same without such additives.
- Adequate volume of handrub to completely wet the hands is crucial.
- Wipes for hand antisepsis are not to be promoted unless a suitable test protocol has been developed; their use should be rejected because of the associated negative impact on the environment.
- If considering an application time shorter than 30 s, handrubs must still pass the unmodified test protocol of EN 1500:2017 within 30 s to be considered safe for use (in Europe).
- Residual moisture on hands will impair the effect of disinfection.

### 5.2 Preoperative surgical-site antisepsis (or disinfection of intact skin)

#### 5.2.1 Perspectives

Standardized surgical-site skin antisepsis must be implemented and rigorously promoted. As mentioned above, alcohols with sustained efficacy by addition of antiseptics are often recommended because of their higher efficacy compared to using alcohol alone with respect to the endpoint prevention of SSI [32], [61], [62], [63], [64], [65], [66]. The application technique should be described in detail. The German KRINKO guidelines on prevention of surgical-site infections published in 2018 recommend application by intensified wiping plus wetting (referred to as “assisted application with soaked applicator” [44], [67]. Before performing surgical-site antisepsis, the patient’s skin should be free of soil, debris, emoilients, cosmetics and alcohol-based products. New applicators must be used for each application.

#### 5.2.2 Conclusions and consensus

There is a consensus among this panel of experts:

- Standardized preoperative skin antisepsis is of paramount importance for the prevention of SSI.
- The optimal contact time is not known. On areas with many sebaceous glands a minimum contact time of 2.5 min is required and application should be performed in two steps:
  1) vigorously rub the skin area using an applicator or sterile forceps and soaked gauze for 30 s,
  2) keep the treated area moist with the antiseptic for at least 2 min (manufacturer’s instructions must be observed).
- For preoperative surgical-site preparation, alcohol-based agents, combined either with chlorhexidine, povidone-iodine or octenidine, are the formulations of choice.

### 5.3 Environmental disinfection

#### 5.3.1 Perspectives

In recent years, there has been more concern for environmental cleaning and disinfection [68], [69], [70], [71], [72], and new recommendations have been published, among them the APSIC Guidelines for environmental cleaning and decontamination (2015) [73], National Guidelines for Clean Hospitals in India (2015) [25], and Canadian Guidelines for Routine Environmental Cleaning of the Operating Room (2017) [74].

Some of the existing earlier guidelines, such as from the KRINKO (2004) [75], [76] or the CDC (2008) [77], are presently under revision or continually updated. In 2016, the German industrial standard organisation DIN established a Working Group, “Hospital Cleaning”, with the objective of defining standardized requirements for cleaning and disinfectant cleaning services in hospitals [78].

The standardization of the disinfectant procedure itself is often insufficiently taken into account, especially in those instances where disinfection is a manual procedure [10]. Five important moments for environmental cleaning and disinfection are:

1. Staff should always consider the cleanliness of high-risk near-patient sites during patient care,
2. before performing aseptic activities,
3. after discharge of patients,
4. after visible surface contamination, and
5. as part of the multi-barrier strategy to control outbreaks.

Apart from training and education as well as compliance of all the different staff members performing and monitoring cleaning and disinfection practices, the challenge is to establish a suitable risk assessment scheme, find a consensus on minimum standards and adapt guidelines to the local situation of each individual healthcare setting in order to devise feasible disinfection protocols [71], [79]. Cleaning and disinfection (bundle) protocols must be based on a holistic concept adapted to local needs. 

Overall guidelines can only provide the framework; they cannot replace the risk assessment and decisions to be made by infection control experts depending on the local situation, not only including epidemiological aspects, but also personnel, organizational, and structural prerequisites. Tools for establishing whether the actual cleaning and disinfection process was effectively performed need to be further developed. Presently, second generation ATP testing solution are available but not in place everywhere. The results should be reported to and discussed with the staff, and further training should take place in order to improve cleaning and disinfection efficacy (cf. the multicenter, randomised trial published by Mitchell et al. on an environmental cleaning bundle [80]). 

Kenters et al. concluded in their recent international survey [48] that global practices in environmental cleaning and disinfection differ widely, and minimum worldwide standards are needed. Routine “cleaning” of patient rooms is most frequently performed with microfiber cloths and mops or with cotton cloths and mops, with 38% of the institutions using detergents only. Terminal cleaning after discharge of patients with multi-drug resistant organisms, carbapenemase-producing *Enterobacteriales* or *C. difficile*, was performed using detergents in 33%, disinfectants (mostly sodium hypochlorite) only in 30%, and both detergents and disinfectants in 37%. As pointed out earlier, halogens are overall the most frequently employed disinfectants. The combination of microfiber cloths with hypochlorite products may, however, greatly reduce the efficacy of cloths. Adequate laundering processes and safe storage of mops and cloths is not always ensured [81]. Monitoring of cleaning and disinfection predominantly occurred by visual inspection.

The optimal cleaning and disinfecting agent for environmental cleaning protocols of the immediate surrounding area of patients colonized or infected with CRE-CRAB-CRPsA has not yet been defined. Three CRE-CRAB-CRPsA studies used hypochlorite (generally a concentration of 1,000 parts per million [ppm]) as an agent to for environmental cleaning [82]. In Germany, good experiences have been reported with peroxides for the disinfection of sink drains, shower drains, and toilets with respect to activity against Gram-negative carbapenemase-producing microorganisms (*M. Exner: personal communication*).

In terms of costs, a stepped-wedge, cluster-randomized trial conducted in 11 hospitals from 6 Australian states confirmed that an evidence-based bundle of cleaning and disinfection measures in a hospital is a cost-effective intervention for reducing the incidence of healthcare-associated infections [83]. 

#### 5.3.2 Conclusions and consensus

There is a consensus among this panel of experts:

- Both environmental cleaning and disinfection are core components in a bundle of infection precautions.
- As a result of their microbicidal mode of action, disinfectant ingredients require a careful benefit-risk assessment to minimize adverse effects on humans and the environment to the greatest extent possible. Consequently, it is not in principle the most effective disinfectant which is to be selected, but the one that is sufficiently effective for the intended purpose, also taking into account the tolerability of the product.
- A risk assessment of the environment to be cleaned and/or disinfected must be performed.
- Hazardous and critical points for disinfection, e.g., frequent-touch points, sink drains (difficult to disinfect), must be defined.
- Wipes must be tested with a suitable test protocol (avoid insufficient concentrations, saturation). Material compatibility of wipe and chemical agent must be included in the testing.
- Criteria for selecting disinfectants with less user acceptance must be explained to the user (e.g., peracetic acid versus quats).
- Outbreak management measures of disinfection must be clearly defined.
- A written protocol and necessary supplies should be available for targeted disinfection in the event of contamination (vomit, stool, blood), including adequate personal protection equipment.
- Management and supervision of patient room cleaning and disinfection must be clearly defined.
- Monitoring and audits of correct disinfection practices for all types of healthcare settings by public health authorities must be mandatory (UV, glow check, ATP second generation), including feedback to the staff.
- Cleaning and disinfection in medical care settings must be acknowledged and promoted as a profession that requires highly skilled personnel (enhance self-esteem of staff) and personnel must be paid accordingly.
- Recruitment and monitoring of outsourced staff must be contractually regulated.
- Enough time must be ensured for cleaning and disinfection.
- Trainers, supervisors and all personnel must be trained according to a curriculum designed by cleaning and teaching professionals and in cooperation with infection control experts.
- Practice-oriented (visual), easy-to-follow training resources must be available.
- Patient (and family) education to reduce bioburden and pay attention to good cleaning and disinfection must be part of the hygiene protocol.

### 5.4 Reprocessing of medical devices and equipment disinfection

#### 5.4.1 Perspectives

After undergoing a reprocessing procedure, a medical device must be safe for use in another patient and may not pose a health hazard due to infectious, pyrogenic, allergenic or toxic reactions or as a result of altered technical-functional properties of the device. Before purchasing a medical device, it is necessary to obtain specific information on the adequate reprocessing procedure for the device, in order to ensure its practicability on site. Unfortunately, the information provided by the manufacturer is often incomplete. Another problem is the recommendation of unsuitable disinfectant agents, which may cause health risks for the patients as a result of toxic residues or insufficient spectrum of activity. Any deviation from the recommendations given by the manufacturers must be substantiated and documented. In Germany, in the event of incomplete or incomprehensible instructions for reprocessing by the manufacturer, the manufacturer is requested to complete, specify and/or correct the information. In individual cases, violations may have to be reported to the Federal Institute for Drugs and Medical Devices (*Bundesinstitut für Arzneimittel und Medizinprodukte*, BfArM).

Transmission of pathogens via incorrectly reprocessed, contaminated medical devices has been a known risk for infection for quite some time [84], [85], [86], [87], [88]. In many countries, the procedures necessary for reuse of medical devices are described in detailed guidelines and recommendations [APSIC, CDC, KRINKO, WHO]. The Spaulding classification of medical devices into the three categories – non-critical, semi-critical, and critical – has been almost universally adopted for the classification of instruments according to their inherent infection risk and is used as a basis for decisions on the reprocessing procedure to be selected. In most countries, manufacturers of medical devices are obliged to provide adequate instructions for reprocessing.

Generally speaking, whenever possible, automated reprocessing is preferred over manual processing methods. However, in practice, not all surgical instruments are suitable for automated cleaning, disinfection and sterilisation, e.g., flexible endoscopes or ultrasound probes, and many healthcare institutions do not possess the necessary equipment. Recently, FDA published warnings on their website as to the insufficient validation of manual reprocessing methods for certain brands of duodenoscopes and bronchoscopes [89]. In 2019, the British Healthcare Infection Society published a new guidance document on decontamination of intracavity medical devices underlining the considerable potential for infection transmission [90].

An international survey conducted 2015/2016 by The Infection Prevention and Control (IPC) workgroup of the International Society of Antimicrobial Chemotherapy (ISAC) in 39 countries found that most facilities (82%) had standard operating procedures for processing flexible endoscopes, with manual cleaning and automatic disinfection being viewed as the most important steps. 50% of the respondents expressed their concern that regular training and education of reprocessing practitioners are needed to improve patient safety [91].

In their multi-society guideline on reprocessing flexible GI endoscopes (2016/17), the U.S. Reprocessing Guideline Task Force recommends: *“The selection and use of disinfectants in the healthcare field is dynamic, and products may become available that were not in existence when this guideline was written. As newer disinfectants become available, persons or committees responsible for selecting disinfectants for GI endoscope reprocessing should be guided by FDA clearance of these products and by information in the scientific literature*” [92]. This statement pinpoints the importance of regulatory bodies as well as state-of-the-art practice taking into account current scientific literature. 

Another topic of special interest is the reprocessing of single-use devices (SUD), i.e., devices designed to be used only once on a single patient. In many countries, reprocessing of SUD is strictly forbidden. Others have regulations with provisions for single-use reprocessing. In some countries it is common practice to reprocess single-use items without standardized protocols or regulations, or despite prohibition by law [93], [94]. 

In 2017, the European Medical Device Regulation (MDR) has set forth requirements for single-use device reprocessing (§17) which can be translated into national law. It basically states that the same demands must be placed on a reprocessed single-use device as on any new device. Therefore, healthcare facilities must make sure that written policies exist, e.g., regarding the type of device which can be reprocessed, the applicable reprocessing procedure, the validation of this procedure, and the number of times a device can be reprocessed [17]. In India, although no up-to-date national policies exist as to the reuse of SUD, a guidance document on reuse of cardiovascular catheters and devices was published as a consensus document in 2017 [95]. 

#### 5.4.2 Conclusions and consensus

There is a consensus among this panel of experts:

- The prerequisite for reprocessing is the risk assessment and classification of medical devices in the categories non-critical, semi-critical, and critical with differing demands on reprocessing.
- The reprocessing procedure for medical devices must be validated. This does not apply for non-critical devices.
- The reprocessing procedure for medical devices must be available as a standard operating procedure and describe the following steps: pre-cleaning (if applicable), cleaning (possibly two times), disinfection, drying, functional testing, packaging, sterilization (if applicable), labelling, release, transport, storage of sterile supplies.
- Automated procedures are preferred over manual reprocessing.
- Reprocessing of single-use medical devices should be regulated in up-to-date (national) guidelines.

### 5.5 Water disinfection

When developing a global strategy for the use of chemical disinfectants in healthcare settings, disinfection of drinking water (and waste water processing) is also part of the disinfection bundle. A large number of reports exist on outbreaks caused by waterborne pathogens [96]. Recent examples include a global outbreak of severe Mycobacterium chimaera disease after cardiac surgery [97], or outbreaks of waterborne *P. aeruginosa* infections in hospitals [98], [99], [100], [101]. In other fields of application, disinfection protocols are also needed for the treatment of hospital water systems and other reservoirs of waterborne pathogens, such as sinks, sink drains, and shower drains.

The standard chemical disinfectant for drinking water production is chlorine, with chlorine dioxide increasingly being considered as an alternative [102], [103]. Other chemical agents frequently used for this purpose are ozone or chloramines. Limited efficacy against protozoan pathogens (in particular Cryptosporidium) and some viruses, a potential tolerance of *L. pneumophila* (for instance) to chlorines after long-term chlorination of hot water distribution systems [102], [104], as well as by-product formation and corrosion effects must be taken into consideration when selecting a suitable chemical disinfectant [105], [106]. For the disinfection of sinks, sink drains and shower drains, peroxides or chlorine are the disinfectants of choice.

Unresolved issues include whether and how to treat hospital sewage in view of the fact that MDRO are found in rivers. Catchment areas of hospitals seem to be an important reservoir for shedding MDRO. 

Disinfection, of course, is not the only means of minimizing infection risks by waterborne pathogens. Other preventative measures include engineering and design solutions for the fittings, pipes, and equipment used with water (such as washer-disinfectors, heater-cooler units). 

#### 5.5.1 India – safe water first

The significance of water safety becomes even more important in countries where drinking water is an extremely rare and valuable resource. In India, the percentage of deaths attributed to inadequate sanitation was estimated to be 9.2% of all deaths in 2006 [107]. Consequently, for India, the number one priority is the availability of safe water throughout all of India. 

The nation-wide campaign “Clean India” – Swach Bharat Abhiyan (SBA) – entails the building of approximately 110 million toilets to eliminate open defecation. The pledge is to have an “open-defecation-free” India by October 2019. In addition, the Ministry of Urban Development published Standard Operating Procedures for Hospitals (Swachh Hospitals) which describe infrastructure norms, assessment and inspection procedures, checklists, and best practice for sanitation and waste managements [108].

This is in line with the Sustainable Development Goal 6 that availability and sustainable management of water and sanitation must be ensured for all. The target for 2030 is to achieve universal and equitable access to safe and affordable drinking water for all. Today, at least 1.8 billion people rely on water sources that are faecally contaminated [102]. WHO points out that disinfection should not be compromised in attempting to control disinfection by-products. However, although chemical disinfection of water which is already faecally contaminated may reduce the hazard of infection, it does not necessarily render it safe [102], [109]. 

#### 5.5.2 Conclusions and consensus

There is a consensus among this panel of experts:

- Waterborne pathogens are a major risk for infections in healthcare settings.
- Rules and regulations for the treatment of hospital sewage need to be in place.
- Facultative pathogens in water distribution systems should be monitored.
- The standard disinfecting agent for plumbing systems is chlorine.
- In some countries, e.g., Germany, point-of-use filtration is seen as the method of choice.
- Sink and shower drains are an important underestimated reservoir. Chlorine or peroxide are the disinfectants of choice here.

## 6 Disinfection practices in agricultural settings

### 6.1 General remarks

Overall, large quantities of disinfectants are used in agriculture, especially liquid surface disinfectants, although formaldehyde fumigation is also common (also see standard operating procedures for animal quarantine and certification services in India [110]). In a market report for 2015, quaternary ammonium compounds and phenols were identified as the prevailing substances for this type of use, followed by oxiziding agents and aldehydes [111]. 

Livestock farms have a higher consumption of disinfectants than grain and produce farms [111]. However, as a consequence of higher demands not only for meat products but also for (greenhouse/horticulture) vegetables and crops, the usage of (“bio-based”) disinfectants is forecast to continue to grow considerably. This includes farms employing “protected” or “organic farming” methods. Aquaculture establishments and equipment should also be taken into consideration for a one-health approach for antimicrobial (disinfection) strategies. The World Organisation for Animal Health issues a Terrestrial Animal Health Code and the Aquatic Animal Health Code [112], [113], which includes standards for the use of antimicrobial agents and also specifies disinfection practices of (aquaculture) establishments and equipment (cf. Section 4 and 6 of the Animal Code [112]). 

### 6.2 Stall and barn disinfection practice in Germany 

Requirements for surface disinfectants to be used in veterinary medicine, food hygiene, or agriculture naturally differ from those to be used in healthcare settings. According to the European Biocidal Products Regulations, they are allocated to PT (product type) 3 “Veterinary Medicine” and product type 4 “Food and Feed area”. Test methods are EN methods and DVG guidelines (German Veterinary Society), respectively, where EN methods for phase 2/step 2 are not yet available. Disinfectants to be used for examination, operation and treatment of animals in (resident) veterinary practices are considered PT 2 products according to the Guidance Document on the European BPR [3], [4]. 

Crowding, transport, and biofilms with high loads of proteins and fats in soil are some of the challenges for stable cleaning and disinfection. Dirt must be removed with suitable cleaning agents before the actual disinfection process. Disinfectants are applied with pressure washers or a foam lance. Attention must be paid to ventilation, which must be turned off during the procedure, and temperature, as the active ingredients often lose their effect below 15°C. It is important to bear in mind that cleaning and disinfection measures need to be evaluated according to animal species via practice-oriented research. Following a risk assessment approach, critical control points in stalls and barns can be identified. For pig facilities, these include, among others, nipple drinkers and troughs. 

The presence of antibiotic-resistant organisms in livestock, e.g., pigs, cattle and poultry, as well as pets, has been well documented [114], [115], [116], [117], [118], [119]. ESBL-producing *Enterobacteriaceae* (ESBL-E) and MRSA have been described to pose a problem and put farmers at an increased risk of colonization [120], [121], [122], [123]. Entry of multidrug-resistant organisms in the food chain (e.g., meat, dairy products) has also been observed [124], [125]. Schmithausen et al. were able to prove that a decontamination protocol in two steps (cleaning and disinfection) of the stalls/barns can lead to a successful elimination of ESBL-E and MRSA in pigs and facilities on a long-term basis [126], [127], but does not prevent acquisition of new MRSA strains.

### 6.3 Conclusions and consensus

There is a consensus among this panel of experts:

- Livestock (and pets) can transmit (multidrug-resistant) microorganisms to humans along the food chain.
- Disinfection can prevent colonization with multidrug-resistant microorganisms.
- The correct choice of disinfectant is crucial, high loads of proteins and fats must be taken into consideration.
- In addition to incorrect dosage and incorrect (shortened) exposure time, the temperature in stalls/barns must also be taken into account, because organic acids and aldehydes in particular are insufficiently effective at temperatures below 10°C.
- In a one-health approach for strategies in chemical disinfection in healthcare settings, disinfectants

## 7 Chemical disinfection from the perspective of emerging and newly identified health risks

### 7.1 Resistance to antimicrobial agents

In 2009, the Scientific Committee on Emerging and Newly Identified Health Risks (SCENIHR) published an opinion paper on the Assessment of the Antibiotic Resistance Effects of Biocides [128]. Since then, the topic of a possible correlation between biocide use and antibiotic resistance has been widely discussed. In 2017, the EU Commission set up an Antimicrobial Resistance (AMR) One Health network of government experts from the human health, animal health, and environmental sectors, as well as the EU scientific agencies working in the human and animal health sectors (ECDC, EMA, and EFSA). This network launched an “EU AMR Action Plan” [129]. While there is no doubt that it is possible to induce resistance to disinfectants in vitro, the authors of this paper believe that more studies are needed on the situation in situ. 

The difficulty of this field of research is aggravated as different vocabulary and definitions are used: What exactly is resistance, co-resistance, tolerance, decreased sensitivity? What about biofilms and VBNC (viable but not culturable) microorganisms? In an opinion paper, Kim and Wood offer the explanation that *„the metabolically active cell population should more accurately be considered tolerant cells, while the dormant cells are the true persister population*“ [130]. Persisters are the remaining, genetically unaltered population of bacterial cells which survive prolonged antibiotic treatment after an initial die-off, with a basically unchanging or slowly decreasing population density due to their lack of metabolic activity. Tolerant cells grow prior to antibiotic addition and then survive longer than exponentially growing cells in the presence of the antibiotic, but their population usually continues to decrease appreciably and the phenotype is a population-wide phenomenon. According to Cohen et al. persistence is an actively maintained state triggered and enabled by a network of intracellular stress that can accelerate processes of adaptive evolution [131]. Hartemann points out that various papers demonstrate that this is possible with disinfectant agents; fewer papers describe a simultaneous antibiotic resistance of these cells [132].

Up to now, demonstration for the emergence of antibiotic and/or disinfectant-resistant organisms in the general environment has been scarce, but in recent years, there has been an increasing number of studies and publications in this field. The occurrence of antibiotic-resistant organisms in various aquatic habitats has been described for almost all parts of the world [133], [134], [135], [136], [137].

### 7.2 New aspects of toxicity: disrupting effects on endocrines

It has to be noted that biocidal products can be found in relevant concentrations in the environment, but that about 60% of “prioritized biocidal products” are not appropriately monitored because of the lack of adequate methodology ([132], results of a workshop on environmental monitoring of biocides in Europe – from prioritization to measurements, Berlin 2012). As mentioned above, data of production and amounts of biocidal products used are lacking.

A relatively new field of research is the investigation of endocrine disruptor (ED) effects of some biocides. An endocrine disruptor is a substance which meets all of the following criteria (according to ECHA):

- It shows and adverse effect in an intact organism or its progeny
- It has an endocrine mode of action, that is, it alters the function(s) of the endocrine system
- The adverse effect is a consequence of the endocrine mode of action

The new endocrine disrupter criteria for biocidal products have been in place since 7 June 2018 under EU Regulation No 528/2012 [138], [139]. This means for Europe, that new biocidal products and biocidal products currently going through the authorization process will need to have an endocrine disrupter assessment included as part of their application. For biocide assessments submitted after 1 September 2013, the evaluating authority may request new information for endocrine disrupter evaluation. Attention must also be paid to non-active substance with ED properties which individual products of a product family may contain. 

### 7.3 Consensus and conclusions

There is a consensus among this panel of experts:

- Researchers should agree on the use of specific vocabulary to describe resistance, tolerance, persistence.
- Prudent use of both antibiotics and disinfectants at the proven-effective concentration is crucial.
- Endocrine disrupting effects of disinfectants have to be further investigated in wildlife and humans with respect to a potential cocktail effect.

## 8 Alternatives to chemical disinfection

Alternatives to liquid chemical procedures such as vaporization with hydrogen peroxide are being promoted for certain applications, such as enhanced room disinfection in the presence of multi-resistant organisms or bacterial spores [140], [141]. However, this method does not represent a substitute for routine chemical disinfection practices and can only be used on visually clean surfaces.

Other approaches entail use of ultraviolet light (UV-C disinfection robots), of cold atmospheric plasma, and the development of antimicrobial coatings (AMC) for frequently-touched surfaces with a large variety of materials such as silver, copper, titanium, zinc, chitosan [142], [143], [144], [145], [146]. An opinion paper initiated by the EU COST Action network (European Cooperation in Science & Technology) for antimicrobial coating innovations published in 2017 came to the conclusion that beneficial and adverse effects of AMC have not yet been fully assessed and more (proactive) research is needed with regard to (eco)toxicological risks, qualtity, efficacy and safety before the use of AMCs in healthcare settings [147].

## 9 Conclusions

Disinfection is one core element in the bundle of infection precaution measures. Disinfection is a process which entails much more than the selection of an active substance. Therefore, a practical guideline to reduce the risk of hospital-acquired infections for hospitals is necessary. Just as compliance was increased by the “Five Moments” of hand hygiene, the “Five Moments” of surface disinfection can improve compliance ([10], cf. chapter 5.3). This analogy corroborates the principle of a balanced relationship between hand hygiene and cleanliness of hand-touch sites, and neither should be prioritized over the other [10], [148].

Very recent reports on a multidrug-resistant, community-associated methicillin-resistant Staphylococcus aureus strain shows the dissemination and ongoing endemic transmission from the Indian subcontinent for Gram-positive bacteria which had hitherto been uncommon. This emphasizes the realization that microorganisms and communicable diseases will never cease to be a challenge for healthcare systems throughout the world [149]. 

It is high time to come to an international agreement on the critical aspects for drawing up a strategy for disinfection and antisepsis in healthcare. As with antibiotics, the use of disinfectants and antiseptics cannot be viewed as an isolated practice in only one sector. 

If we want to continue to rely on effective substances and procedures to protect us from infection, we need a holistic One-Health-Approach and prudent use of both antibiotics and disinfectants at proven effective concentrations. Independent research on existing methods for disinfection and novel methods for the reduction of pathogens to a safe level must be promoted in all parts of the world. And as with safe water for all, the availability and affordability of quality-assured active substances for infection prevention must be ensured for all people everywhere.

General aspects to be taken into considerations when developing a global strategy for disinfection and antisepsis are as follows.

**Holistic approach**

- Involve international professional associations in order to develop common sustainable, global strategies for disinfection of animate and inanimate surfaces.
- Have a global glossary and definition of key terms for cleaning and disinfection.
- Take into consideration overall strategies and novel approaches to reduce bioburden, transmission paths and biofilm formation (by suitable design of devices, architecture, surface finishes, ventilation etc).
- Adopt a one-health approach.
- Be aware of the local epidemiological situation and health status depending on demographics.
- Be aware of pathogen reservoirs (including MDRO) in the environment.
- View cleaning, disinfection and antisepsis as constituents of a (chemical) barrier bundle to prevent the transmission of pathogenic agents.
- Develop a strategy for the treatment of hospital sewage, especially for sink drains, shower drains, and toilets.

**Agents for disinfection**

- Be aware of where, how, and by whom substances for disinfection are produced.
- Establish a checklist for selecting disinfectants.
- Have an open-access positive list of disinfectants with their intended use/application in healthcare.
- Have an open-access database of microbicidal substances which states, e.g., the overall quantity produced, the quantity used in specific sectors (healthcare, consumer, others), other substances and by-products frequently used in combination, materials’ compatibility, potential health or environmental hazards.
- Ensure world-wide availability of quality-controlled effective products at low cost.

**Disinfection Practice **

- Establish a framework for risk assessment and defining critical control points for disinfection in medical and non-medical settings for all applications of antisepsis/disinfection [10], [35].
- Establish a framework for requirements for the effective performance of (cleaning and) disinfection procedures.
- Provide suitable tools and curricula for training and education of effective standardized cleaning and disinfection practices for all staff performing this task.
- Allow sufficient time for cleaning personnel to clean and disinfect.
- Take occupational hazards into consideration and inform about safety precautions for each product.
- Monitor the complete procedure of disinfection, including, e.g., processing of wipes/mops or effectiveness of wipes with certain substances.
- Provide performance feedback to staff.
- Establish an outcome surveillance system.
- Require audits for cleaning and disinfection performed by public health authorities.
- Include patients and visitors in hospitals and other medical institutions as well as the general public in concepts of cleaning and disinfection.
- Include a behavioral psychologist in the infection control team and establish patient safety culture [150].

## Notes

### Competing interests

Peter Goroncy-Bermes, Wolfgang Merkens, Peter Oltmanns and Kathrin Steinhauer are employees of Schülke & Mayr GmbH, Norderstedt, Germany, a producer of disinfectants. The other authors declare that they have no competing interests.

## Supplementary Material

Examples of definitions for disinfection and disinfectants

## Figures and Tables

**Table 1:**
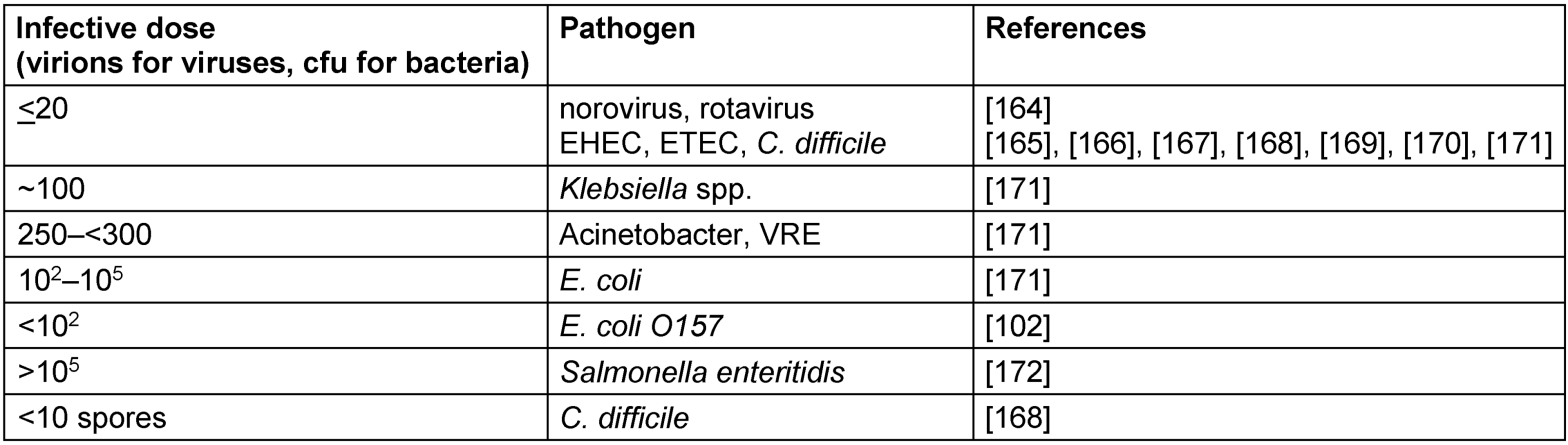
Indicative values for infective doses of various pathogens

**Table 2:**
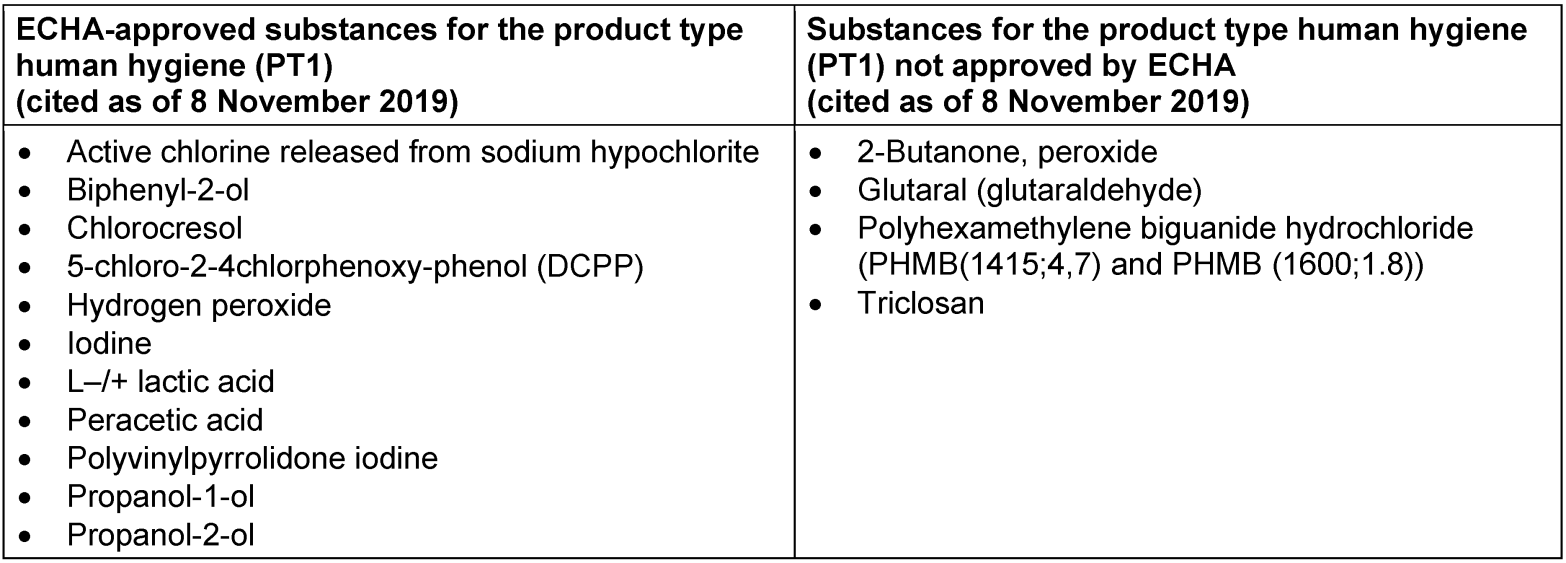
Approved substances for product type 1 according to ECHA

**Table 3:**
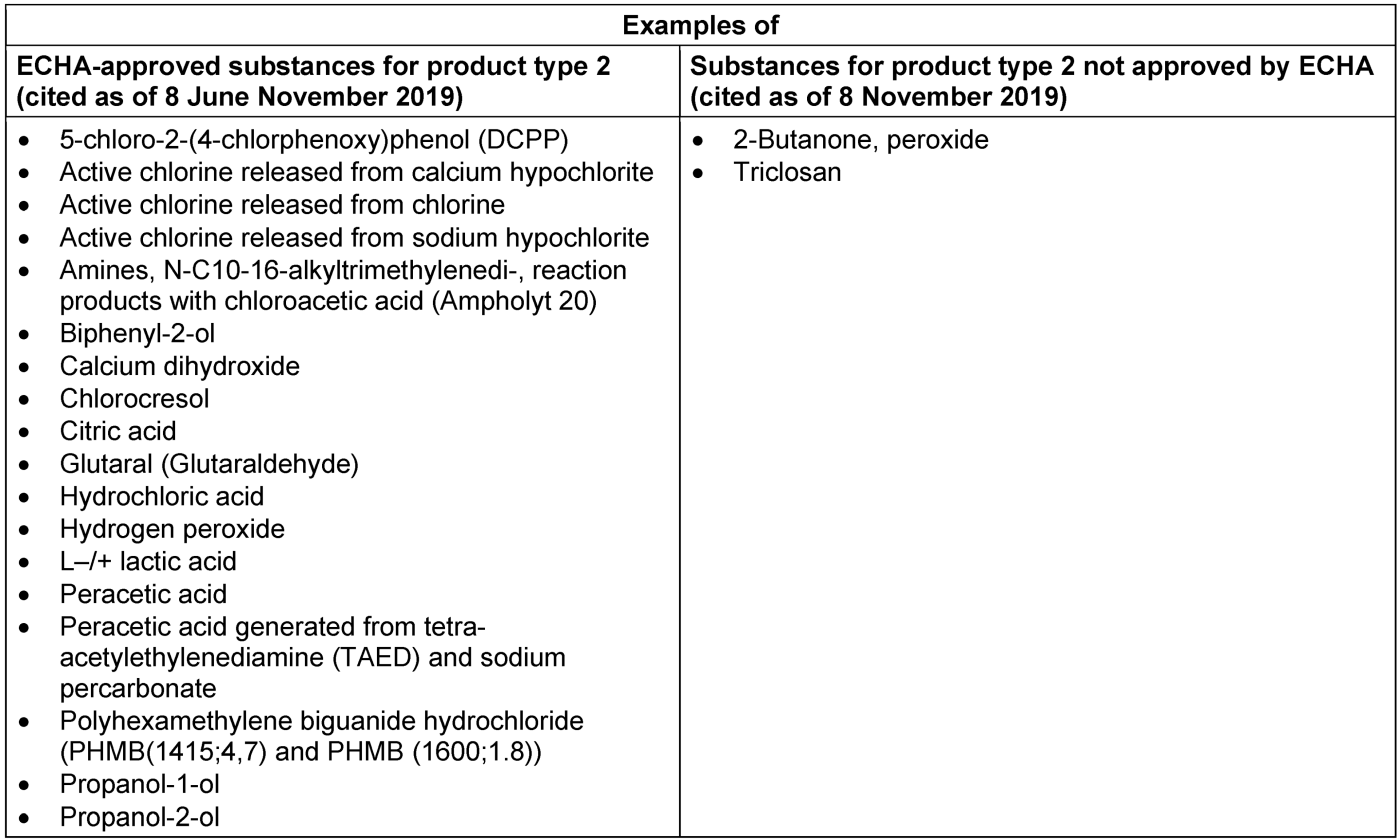
Approved substances for product type 2 according to ECHA

**Table 4:**
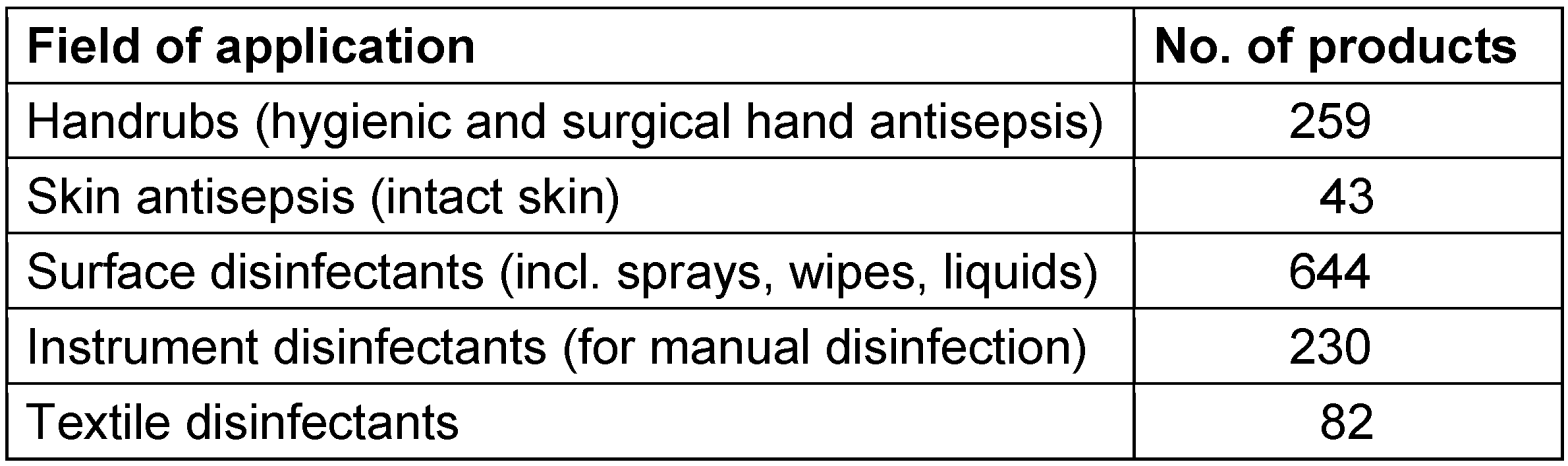
List of products per category (field of application) in the VAH List of Disinfectants ([8] as of 1 October 2019)

**Table 5:**
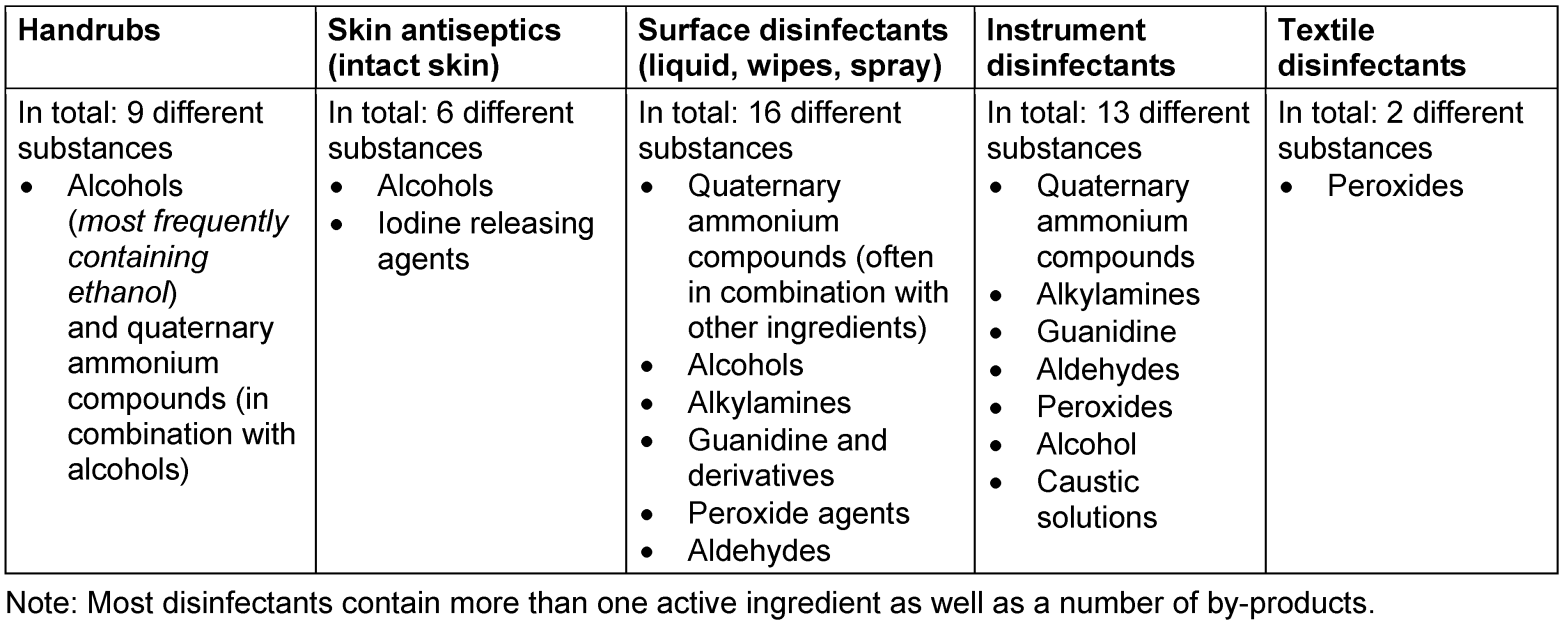
Main substances per category (as of 1 October 2019) in the VAH List of Disinfectants [8]
