# Supplementary material for: Chemical disinfection in healthcare settings: critical aspects for the development of global strategies
Source: GMS Hyg Infect Control. 2020 Dec 23;15:Doc36. doi: 10.3205/dgkh000371 (PMC7818848; doi:10.3205/dgkh000371)
Supplement: Examples of definitions for disinfection and disinfectants [file HIC-15-36-s-001.pdf]

Attachment 1: Examples of definitions for disinfection and disinfectants and categorization of disinfectants by regulatory bodies and hospital infection-control professional societies
See references in the article for full references.

Reference/source	Definition of disinfection<br>(or antisepsis)	Definition of disinfectant<br>(or antiseptic)	Categories, classification or types of disinfectants (and/or antiseptics) described
**Wikipedia**<br>https://en.wikipedia.org/wiki/Disinfectant<br>**[151]**	**Disinfection** does not necessarily kill all microorganisms, especially resistant bacterial spores; it is less effective than sterilization, which is an extreme physical and/or chemical process that kills all types of life.	**Disinfectants** are antimicrobial agents that are applied to the surface of non-living objects to destroy microorganisms that are living on the objects (retrieved from Infection Control Glossary, Division of Oral Health, U.S. Centers for Disease Control; 2016).	E.g.<br>• air disinfectants<br>• alcohols<br>• aldehydes<br>• home disinfectants<br>• non-chemical
**WHO Guideline Hand Hygiene (2009)**<br>**[35]**	Hand **disinfection** is extensively used as a term in some parts of the world and can refer to antiseptic handwash, antiseptic handrubbing, hand antisepsis/decontamination/degerming, handwashing with an antimicrobial soap and water, hygienic hand antisepsis or hygienic handrub.<br><br>Since disinfection normally refers to the decontamination of inanimate surfaces and objects, this term is not used in these Guidelines.<br>(page 2)	(Disinfectant: not specifically defined)	• alcohol-based (hand)rub<br>• antiseptic agent<br>• antiseptic hand wipe<br>• waterless antiseptic agent
**WHO and Pan American Health Organization: Decontamination and Reprocessing of Medical Devices for Healthcare Facilities (2016)**<br>**[47]**<br>Glossary	**Disinfection** is a process to reduce the number of viable microorganisms to a less harmful level. This process may not inactivate bacterial spores, prions and some viruses. (Glossary, page 10)<br><br>Levels of decontamination, disinfection: The destruction or removal of microorganisms at a level that is not harmful to health and safe to handle. This process does not necessarily include the destruction of bacterial spores<br>(Table 1, page 22).	**Disinfectant** is a chemical agent that is capable of killing most pathogenic microorganisms under defined conditions, but not necessarily spores. It is a substance that is recommended for application to inanimate surfaces to kill a range of microorganisms. The equivalent agent, which kills microorganisms present on skin and mucous membrane, is called an antiseptic<br>(Glossary, page 10).	Types of commonly-used chemical disinfectants described by active substance<br>(Table 9, pp. 70)<br><br>Levels of disinfection (Figure 19, p. 79) by germicidal activity<br>• low level<br>• intermediate level<br>• high level

Attachment 1 to: Exner M, Bhattacharya S, Gebel J, Goroncy-Bermes P, Hartemann P, Heeg P, Ilschner C, Kramer A, Ling ML, Merkens W, Oltmanns P, Pitten F, Rotter M, Schmithausen RM, Sonntag HG, Steinhauer K, Trautmann M. Chemical disinfection in healthcare settings: critical aspects for the development of global strategies. GMS Hyg Infect Control. 2020;15:Doc36. DOI: 10.3205/dgkh000371

Reference/source	Definition of disinfection (or antisepsis)	Definition of disinfectant (or antiseptic)	Categories, classification or types of disinfectants (and/or antiseptics) described
**ISO 15883-1:2006 Washers-Disinfectors Part 1: General requirements, terms and definitions and tests [152]**	**Disinfection**: reduction of the number of viable microorganisms on a product to a level previously specified as appropriate for its intended further handling or use.<br><br>**Chemical disinfection**: achieved by the action of one or more chemicals, the primary purpose of which is to be microbicidal.	(Disinfectant: not specifically defined)	
**EU Regulation No. 528/2012 (Biocide Regulation) [3]**	(Disinfection: not specifically defined)	(Disinfectant: not specifically defined)<br>**Biocidal product** means:<br>• any substance or mixture, in the form in which it is supplied to the user, consisting of, containing or generating one or more active substances, with the intention of destroying, deterring, rendering harmless, preventing the action of, or otherwise exerting a controlling effect on, any harmful organism by any means other than mere physical or mechanical action;<br>• any substance or mixture, generated from substances or mixtures which do not themselves fall under the first indent, to be used with the intention of destroying, deterring, rendering harmless, preventing the action of, or otherwise exerting a controlling effect on, any harmful organism by any means other than mere physical or mechanical action.<br><br>A treated product that has a primary biocidal function shall be considered a biocidal product (Article 3, Definitions (a), L 167/9).<br>"Active substance" means a substance or a micro-organism that has an action on or against harmful organisms;<br>(Article 3, Definitions (c), L 167/10)	**Biocidal product-types (PT), main goup 1: disinfectants**<br>Classified according to their intended use:<br>• PT1: human hygiene<br>• PT2: disinfectants and algaecides not intended for direct application to humans or animals<br>• PT3: veterinary hygiene<br>• PT4: food and feed area<br>• PT5: drinking water<br>(Annex V, L 167/105)

Reference/source	Definition of disinfection (or antisepsis)	Definition of disinfectant (or antiseptic)	Categories, classification or types of disinfectants (and/or antiseptics) described
**ECHA Guidance on the Biocidal Products Regulation Volume II Efficacy – Assessment and Evaluation Parts B+C Version 3.0 (April 2018) [4]**	**Skin disinfection within PT1:** Skin disinfection is the reduction of the number of micro-organisms on skin, achieved by the irreversible action of a product, to a level judged to be appropriate for a defined purpose.<br>**PT2, 3, 4**: **disinfection** is the reduction of the number of micro-organisms in or on an inanimate matrix - achieved by the irreversible action of a product, to a level judged to be appropriate for a defined purpose.	A **disinfectant** is a product that reduces the number of micro-organisms (fungi, yeasts, viruses, algae, bacteria or spores) in or on an inanimate matrix – achieved by the irreversible action of a product, to a level judged to be appropriate for a defined purpose.	Examples for PT1 and PT2:<br>PT1<br>• mainly hand disinfectants (p. 61)<br>PT2<br>• disinfectants for hard surfaces (including wipes)<br>• disinfectants for soft furnishings<br>• room disinfection with vaporized biocide<br>• disinfectants for swimming pools, spas and hot tubs<br>• toilets (in various environments)<br>• air-conditioning systems<br>• equipment or instrument disinfection by immersion<br>• textiles<br>• biofilm disinfection<br>• soil (pp. 65ff)
**EN 14885:2018 Chemical disinfectants and antiseptics CEN/TC 216 Application of European Standards for chemical disinfectants and antiseptics (2018) [153]** (Definitions/Glossary)	**Antisepsis:**<br>application of an antiseptic on living tissues causing an action on the structure or metabolism of microorganisms to a level judged to be appropriate to prevent and/or limit and/or treat an infection of those tissues.<br>**Chemical disinfection:**<br>reduction of the number of microorganisms in or on an inanimate matrix, achieved by the irreversible action of a product on their structure or metabolism, to a level judged to be appropriate for a defined purpose.<br>Scopes of CEN/TC 216:<br>• WG1 human medicine<br>• WG2 veterinary use<br>• WG3 food hygiene and domestic and institutional use<br>*(Note: requirements on the exact log reduction are laid down in the EN Standards according to CEN/TC 216 scope, field of application and activity spectrum)*	**Antiseptic:**<br>product – excluding antibiotics – that is used to bring about antisepsis.<br>**Disinfectant**:<br>product that is capable of chemical disinfection.<br>Preoperative antiseptic agents are not included in the scope of this standard.	Categories of disinfectants in WG1:<br>• hygienic handrub<br>• hygienic handwash<br>• surgical handrub<br>• surgical handwash<br>• surface disinfection (solid surface which cannot be immersed) with or without mechanical action<br>The application includes, e.g., circulation, dipping, flooding, spraying, fogging, wiping etc.<br>• instrument disinfection (surface disinfection by immersion)<br>• textile disinfection<br>Note: The same categories are used by the **VAH (Association for Applied Hygiene)** in Germany

Reference/source	Definition of disinfection (or antisepsis)	Definition of disinfectant (or antiseptic)	Categories, classification or types of disinfectants (and/or antiseptics) described
**Kommission für Krankenhaus-hygiene und Infektionsprävention: Hygienic Requirements for Cleaning and Disinfecting Surfaces (2004)**<br>(Germany)<br>**[76]**	**Disinfection** is a process which reduces the count of viable microorganisms on an item/an area as a result of destruction/inactivation – confirmed by standardized quantifiable proof of efficacy – so that it no longer poses an infection risk.<br>Differentiation between:<br>• routine disinfection (prophylactic disinfection)<br>• targeted disinfection (e.g., visible contamination, terminal disinfection, outbreak situations, occurrence of specific pathogens)	Note:<br>According to KRINKO 2004:<br>Cleaning and disinfection carried out as a one-step process:<br>"**one-step disinfectant cleaners**" must be specifically declared as suitable for this purpose by the manufacturer.	• surface disinfectants<br>• one-step disinfectant cleaners
**EPA Pesticide Registration**<br>(U.S.A.)<br>**[154]**	(Disinfection: not specifically defined)	EPA regulates **antimicrobial pesticides**.<br>An antimicrobial pesticide is intended to disinfect, sanitize, reduce, or mitigate growth or development of microbiological organisms or protect inanimate objects, industrial processes or systems, surfaces, water, or other chemical substances from contamination, fouling, or deterioration caused by bacteria, viruses, fungi, protozoa, algae, or slime.<br><br>**Disinfectants:** Used on nonliving surfaces and objects to destroy or irreversibly inactivate infectious fungi and bacteria, but not necessarily their spores.<br><br>**Germicide:** A germicide is a substance or mixture of substances that kill a number of microorganisms (e.g., viruses, fungi and bacteria).	Types of antimicrobial products:<br>• Non-public-health products<br>• Public health products:<br>○ sterilants,<br>○ sporicides,<br>○ disinfectants,<br>○ sanitizers<br><br>Two major types of disinfectants:<br>1. Hospital type disinfectants for infection control, used on:<br>○ Medical and dental instruments<br>○ Floors<br>○ Toilet seats, and other surfaces.<br>2. General use disinfectants, used in:<br>○ Households<br>○ Swimming pools<br>○ Water purifiers<br>(disinfectants can be approved for both uses)

Reference/source	Definition of disinfection (or antisepsis)	Definition of disinfectant (or antiseptic)	Categories, classification or types of disinfectants (and/or antiseptics) described
**EPA**<br>**OCSPP 810.2200 Disinfectants for Use on Environmental Surfaces – Guidance for Efficacy Testing**<br>[EPA 712-C-17-004]<br>(Feb 2018**)**<br>(U.S.A.)<br>**[19]**	(Disinfection: not specifically defined)	**Hospital or healthcare disinfectants**: products recommended for use in hospitals, clinics, dental offices, nursing homes, sickrooms, or any other healthcare-related facility.	Basic efficacy claims for disinfectants:<br>• Limited spectrum disinfectant/hard non-porous surfaces<br>• Broad spectrum disinfectant/hard non-porous disinfectant (active against Gram-positive and Gram-negative bacteria)<br>• Hospital or healthcare disinfectant/hard non-porous surfaces.<br><br>Additional disinfectant activity claims are possible (additional bacteria, fungicidal, virucidal, tuberculocidal) as well as<br>• Internal toilet and urinal bowl surfaces<br>(Table 1, page 2)
**CDC**<br>**Guideline for Disinfection and Sterilization in Healthcare Facilities**<br>Definition of Terms<br>(2008, update May 2019)<br>(U.S.A.)<br><br>**[77]**	**Disinfection** describes a process that eliminates many or all pathogenic microorganisms, except bacterial spores, on inanimate objects. In health-care settings, objects are usually disinfected by liquid chemicals or wet pasteurization. […]<br>Unlike sterilization, disinfection is not sporicidal.<br>(pp. 9 and 10)	**Antiseptics** are germicides applied to living tissue and skin.<br><br>**Disinfectants** are antimicrobials applied only to inanimate objects.<br>A few disinfectants will kill spores with prolonged exposure times (3–12 hours); these are called **chemical sterilants**<br><br>The term **germicide** includes both antiseptics and disinfectants.<br>(p. 10)	• *Low-level disinfectants*<br>• *Intermediate-level disinfectants*<br>• High-level disinfectants<br>(pp. 11)
**CDC Glossary**<br>(Website)<br>**Guideline for Disinfection and Sterilization in Healthcare Facilities**<br>**[155]**	(Disinfection: not specifically defined)**-**	***Disinfectant:***<br>*usually a chemical agent (but sometimes a physical agent) that destroys disease-causing pathogens or other harmful microorganisms but might not kill bacterial spores. It refers to substances applied to inanimate objects.*	*EPA-groups of disinfectants by product label*<br>• *limited,*<br>• *general (broad-spectrum),*<br>• *hospital.*<br><br>*Hospital Disinfectant:*<br>*Disinfectant registered for use in hospitals, clinics, dental offices, and any other medical-related facility.*

Reference/source	Definition of disinfection (or antisepsis)	Definition of disinfectant (or antiseptic)	Categories, classification or types of disinfectants (and/or antiseptics) described
**Ministry of Health, Health Canada Guidance Document Disinfectant Drugs** (January 2018) Canada **[156]**	(disinfection: not specifically defined)	**Antimicrobial agent:**<br>Defined in section C.01A.001 of the Food and Drug Regulations as: a drug that is capable of destroying pathogenic micro-organisms and that is labelled as being for use in the disinfection of environmental surfaces or medical devices, as defined by the Medical Devices Regulations, that<br>(a) are not invasive devices as defined in those Regulations; and<br>(b) that are intended to come into contact with intact skin only.<br><br>**Disinfectant**:<br>A substance, or mixture of substances, capable of destroying or irreversibly inactivating pathogenic (disease-causing) and potentially pathogenic (opportunistic) microorganisms, but not necessarily bacterial spores, present on environmental surfaces and inanimate objects due to the antimicrobial action of the active ingredient(s).<br><br>**Disinfectant-sanitizer:**<br>A chemical product represented for use as a sanitizer on hard non-porous environmental surfaces and inanimate objects which is also represented for use as a hard surface disinfectant.	Categories of disinfectants for use on non-critical medical devices and hard non-porous environmental surfaces and inanimate objects by premise: in<br>• domestic,<br>• industrial/institutional,<br>• hospital,<br>• food processing<br>• and/or barn<br>premises.<br><br>• Hospital disinfectants<br>(require a drug identification number (DIN) for sale in Canada)<br><br>Categories by efficacy level<br>• high-level disinfectants<br>• intermediate-level disinfectants<br>• low-level disinfectant<br><br>Types by activity claim (bactericide, virucide etc.)<br>• limited disinfectants
**Ministry of Health, Health Canada Guidance Document – Human-Use Antiseptic Drugs** (November 2009/July 2019) Canada **[157]**	(disinfection: not specifically defined)	Handrubs are not considered disinfectants: human-use antiseptic products for application on the skin<br>• used with water (referred to as washes)<br>• or without water (referred to as rubs),<br><br>Antiseptic skin products include preoperative skin preparations.	The category of professional healthcare use includes:<br>• professional hygienic handrubs,<br>• surgical handrubs,<br>• professional hygienic handwash,<br>• surgical handwash,<br>• patient preoperative skin preparations<br><br>(Chapter 3.1.4, Guidance Document Human-Use Antiseptic Drugs)

Reference/source	Definition of disinfection (or antisepsis)	Definition of disinfectant (or antiseptic)	Categories, classification or types of disinfectants (and/or antiseptics) described
**APSIC:**<br>**Asia Pacific Society of Infection Control Guidelines for Disinfection and Sterilisation of Instruments in Health Care Facilities**<br>(rev. 2017)<br>**[158]**	**Disinfection** refers to a process that eliminates all pathogenic microorganisms, except bacterial spores, on inanimate objects (p. 14).<br><br>Glossary:<br>**Disinfection:** thermal or chemical destruction of pathogenic and other types of microorganisms. Disinfection is less lethal than sterilization because it destroys most recognized pathogenic microorganisms but not necessarily all microbial forms (e.g., bacterial spores) (p. 6).	**Antimicrobial agent:** any agent that kills or suppresses the growth of microorganisms<br><br>**Antiseptic:** substance that prevents or arrests the growth or action of microorganisms by inhibiting their activity or by destroying them. (especially used for preparations applied topically to living tissue)<br><br>**Disinfectant:** usually a chemical agent (but sometimes a physical agent) that destroys disease-causing pathogens. It refers to substances applied to inanimate objects.	Two major categories for disinfection of reusable medical equipment/devices:<br>• high-level disinfection<br>• low-level disinfection<br>(p. 66)<br><br>Methods:<br>• liquid chemical disinfection<br>• pasteurization (hot water disinfection minimum 71°C for 30 min)
**Australian Therapeutic Goods Administration (TGA) Therapeutic Goods Regulations 1990**<br>**[159]**<br><br>**Therapeutic Goods (Standard for Disinfectants and Sanitary Products) TGA Order 104**<br>**(29 March 2019)**<br>replaces the previous TGO 54 Standard for Disinfectants and sterilants<br>**[160]**	(disinfection: not specifically defined)	**Disinfectant** means a substance:<br>(a) that is recommended by its manufacturer for application to an inanimate object to kill microorganisms; and<br>(b) that is not represented by the manufacturer to be suitable for internal use.<br>(TGA Regulations, Part 1, Chapter 2 Interpretation)	TGA Regulations, categories<br>• hospital-grade disinfectant means a disinfectant that is represented to be suitable for therapeutic use (with and without specific claims)<br>• household (and commercial)-grade disinfectant (with or without specific claims)<br>The term 'specific claims' covers virucidal, sporicidal, tuberculocidal, fungicidal or other biocidal activity.<br><br>TGA 104<br>Hospital-grade disinfectants types:<br>• general purpose use on surfaces<br>• surface spray disinfectants<br>• disinfectant wipes or sponges for single use and for multiple use<br>• disinfectant wipes or sponges for single use and for multiple use where disinfectant cannot be expressed

Reference/source	Definition of disinfection (or antisepsis)	Definition of disinfectant (or antiseptic)	Categories, classification or types of disinfectants (and/or antiseptics) described
**Australian Guidelines for the Prevention and Control of Infection in Healthcare**<br>May 2019<br>**[161]**	Glossary, p. 303<br>**Disinfection:**<br>Reduction of the number of viable microorganisms (by physical or chemical means) on a product to a level previously specified as appropriate for its intended further handling or use	**Antiseptic:**<br>Biocide or products that destroy or inhibit the growth of microorganisms in or on living tissue.<br><br>**Alcohol-based hand rub:**<br>A TGA-listed alcohol-containing preparation designed for reducing the number of viable micro-organisms on the hands without the use or aid of running water, and which is listed on the ARTG (Australian Register of Therapeutic Goods) as a medicinal product [162].<br><br>**Disinfectant:**<br>A substance<br>(a) that is recommended by its manufacturer for application to an inanimate object to kill microorganisms<br>(b) that is not represented by the manufacturer to be suitable for internal use.	• alcohol-based hand rubs<br>• surgical hand rub/hand preparation<br>• skin antiseptic<br>• hospital-grade disinfectants with specific claims<br>• hard surface disinfectants with specific claims including hospital, household and commercial grade disinfectants (without specific claims: considered “OTG”, other therapeutic goods)<br>• medical device disinfectants (instrument disinfection):<br>• low level<br>• intermediate level<br>• high level
**Ministry of Health & Family Welfare KAYA KALP. National Guidelines for Clean Hospitals**<br>(India)<br>(2015)<br>**[25]**	**Disinfection:**<br>The inactivation of disease-producing microorganisms. Disinfection does not destroy bacterial spores. Medical equipment/devices must be cleaned thoroughly before effective disinfection can take place.<br>(Glossary of terms)	**Antiseptic:**<br>An agent that can kill microorganisms and is applied to living tissue and skin.<br><br>**Disinfectant:**<br>A product that is used on surfaces or medical equipment/devices which results in disinfection of the equipment/device. Disinfectants are applied only to inanimate objects. Some products combine a cleaner with a disinfectant.<br>(Glossary of terms)	• hospital-grade disinfectant<br>• low-level disinfectant

Reference/source	Definition of disinfection (or antisepsis)	Definition of disinfectant (or antiseptic)	Categories, classification or types of disinfectants (and/or antiseptics) described
**Ministry of Health & Family Welfare NFI: National Formulary of India** (4th edition, 2011) **[23]**	(disinfection: not specifically defined)	An **antiseptic** destroys or inhibits growth of micro-organisms on living tissues without causing injurious effects when applied to surfaces of the body or to exposed tissues. Some antiseptics are applied to the unbroken skin or mucous membranes, to burns and to open wounds to prevent sepsis by removing or excluding microbes from these areas (p. 271).<br><br>A **disinfectant** is a chemical agent, which destroys or inhibits growth of pathogenic micro-organisms in the non-sporing or vegetative state. Disinfectants do not necessarily kill all organisms but reduce them to a level, which does not harm health or the quality of perishable goods. Disinfectants are applied to inanimate objects and materials such as instruments and surfaces to control and prevent infection. They may also be used to disinfect skin and other tissues prior to surgery (see also Antiseptics, above) (p. 275).	
**Market analysis reports, or BCC Research Company survey [163]**	–		**Antiseptics and Disinfectants, by application:**<br>• antiseptics and disinfectants for domestic use<br>• antiseptics and disinfectants for institutional/ commercial use<br><br>**Antiseptics and disinfectants, by type:**<br>• alcohols<br>• aldehydes<br>• iguanides and amidines<br>• iodine products<br>• oxidizing agents<br>• phenol and derivatives<br>• quaternary ammonium compounds<br>• silver compounds<br><br>**Antiseptics** in the **form** of hand washes, hand rinses, sanitizers, gels, soaps, wipes, and scrubs are used on skin, mucosa or cavities to destroy pathogens and/or inhibit their growth.<br><br>**Disinfectants** in the **form** of solutions, powder, sprays, gels, and so forth are used for decontamination of surfaces, equipment, or other inanimate objects.
